# Phosphorylation as a Tool To Modulate Aggregation Propensity and To Predict Fibril Architecture

**DOI:** 10.1002/cbic.201100607

**Published:** 2011-12-15

**Authors:** Nathalie M Valette, Sheena E Radford, Sarah A Harris, Stuart L Warriner

**Affiliations:** 1 School of Chemistry, University of LeedsLeeds, LS21 3DS (UK); 2 Astbury Centre for Structural Molecular Biology, University of LeedsLeeds, LS21 3DS (UK); 3 Institute for Molecular and Cellular Biology, University of LeedsLeeds, LS21 3DS (UK); 4 School of Physics and Astronomy, University of LeedsLeeds, LS21 3DS (UK)

**Keywords:** molecular dynamics, peptides, phosphopeptides, protein folding, self-assembly

## Abstract

**Abstract:**

Despite the importance of post-translational modifications in controlling the solubility and conformational properties of proteins and peptides, precisely how the aggregation propensity of different peptide sequences is modulated by chemical modification remains unclear. Here we have investigated the effect of phosphorylation on the aggregation propensity of a 13-residue synthetic peptide incorporating one or more phosphate groups at seven different sites at various pH values. Fibril formation was shown to be inhibited when a single phosphate group was introduced at all seven locations in the peptide sequence at pH 7.5, when the phosphate group is fully charged. By contrast, when the same peptides were analysed at pH 1.1, when the phosphate is fully protonated, fibrils from all seven peptide sequences form rapidly. At intermediate pH values (pH 3.6) when the phosphate group is mono-anionic, the aggregation propensity of the peptides was found to be highly dependent on the position of the phosphate group in the peptide sequence. Using this information, combined with molecular dynamics (MD) simulations of the peptide sequence, we provide evidence consistent with the peptide forming amyloid fibrils with a class 7 architecture. The results highlight the potential utility of phosphorylation as a method of reversibly controlling the aggregation kinetics of peptide sequences both during and after synthesis. Moreover, by exploiting the ability of the phosphate group to adopt different charge states as a function of pH, and combining experimental insights with atomistic information calculated from MD simulations as pH is varied, we show how the resulting information can be used to predict fibril structures consistent with both datasets, and use these to rationalise their sensitivity of fibrillation kinetics both to the location of the phosphate group and its charge state.

## Introduction

The aggregation of proteins and peptides into amyloid fibrils is associated with a number of life-threatening human disorders,[^1^] including type II diabetes,^[^^2^] and Alzheimer’s^[^^3^] and Parkinson’s diseases.^[^^4^] The monomeric precursors associated with these diseases range from small peptides (Aβ, amylin) to natively unfolded (α-synuclein) and natively folded proteins (β_2_-microglobulin (β_2_M), lysozyme). Despite their lack of sequence identity or structural similarity, the fibrils generated from different proteins share a common cross-β structural motif characterised by hydrogen-bonded β-strands arranged perpendicular to the fibril axis to form β-sheets, which can subsequently stack together to generate a regular assembly.[^5^]

Atomic details of the architecture of amyloid fibrils have been obtained by using a range of techniques, including solid-state NMR spectroscopy (ssNMR),[^6^] cryo-electron microscopy (cryo-EM),[^7^] electron paramagnetic resonance (EPR),[^8^] X-ray fibre diffraction,[^9^] hydrogen/deuterium (H/D) exchange,[^10^] and proline-scanning mutagenesis.[^11^] Despite these developments, it is still a challenge to obtain atomistic details about amyloid structures. To meet these challenges, recent studies have focused on the use of small peptides as model systems to gain atomic details on the organisation of the β-strands and β-sheets within the fibril. X-ray crystallographic analysis of fibrils formed from a range of short amyloidogenic peptide fragments has highlighted a limiting set of eight possible arrangements of β-strands and β-sheets within the cross-β structure of amyloid, referred to as steric zippers because of the tight intermeshing of the β-sheet side chains to form a dry interface within the protofilament structure.[^12^] Although this approach has proved successful in determining the structures of a large number of proteins or peptides, they rely on the ability to crystallise relevant small fragments. Hence, a simple, rapid and reversible chemical approach to gain insight into fibril structure and stability is warranted.

Phosphorylation has been used to modulate the aggregation propensity of proteins and peptides, including tau,[^13^] α-synuclein,[^14^] and peptide model systems.[^15^] The amyloidogenic potential of α-synuclein, for example, can be suppressed, in vitro, at neutral pH by inserting a phosphate group at Ser129; however, mutating the serine residue into an aspartate or a glutamate does not mimic the effect of the phosphate group, despite their structural and electrostatic similarities.[^14^] Phosphorylation has also been used to modulate the aggregation propensity of a coiled coil peptide model by enzymatically triggering a structural switch leading to amyloid formation.[^15b^^,^ ^c^] However, these studies have provided only a phenomenological description of the effect of the addition of a phosphate moiety, and they do not consider in detail either pH or positional dependence or an atomistic description of the observed effects.

In contrast, in this study, we report for the first time, the sequence- and pH-dependence of the effect of phosphorylation on aggregation using a small peptide of the human protein β_2_M corresponding to residues 59 to 71, and by combining experiment and simulation we provide atomistic models for the origin of the effects observed. Although there is no evidence that the full-length protein is phosphorylated, in vivo, this 13-residue sequence (DWSFYLLYYTEFT, known as strand E)[^16^] forms an ideal model system for the studies presented, as six out of the 13 residues can be phosphorylated reversibly, in vitro, and the peptide has been shown to be highly aggregation-prone when using theoretical methods[^17^] and in vitro aggregation studies.[^16^^,^ ^18^] The effect of inserting a phosphate moiety at a specific position within the sequence on fibril formation was then investigated by using thioflavin T (ThT) fluorescence and negative stain transmission electron microscopy (TEM) at pH values corresponding to the different protonation states of the phosphate group. Protofilament structures of the fibrils formed by strand E and its phosphorylated variants consistent with the experimental data were then constructed and their stabilities as a function of pH were monitored by using MD simulations. By combining the experimental and simulation data, we derive a model for the fibrillar architecture of this peptide able to satisfy all the data obtained that contains an antiparallel, shifted organisation of the β-strands organised within a fibril of class 7^12^ architecture.

## Results and Discussion

### Synthesis of peptides

In order to investigate the effect of phosphorylation on the aggregation propensity of the amyloidogenic sequence of β_2_M corresponding to residue 59 to 71 (strand E), a series of tridecapeptides containing zero, one or two phosphoamino acid residues was synthesised by using Fmoc solid-phase synthesis.[^19^] The C terminus was prepared as the amide and the N terminus was capped with an acetyl group. Significant optimisation of the synthesis was required as high loading resins led to incomplete amino acid coupling when the core amyloidogenic sequence “FYLLYY” was reached. Synthesis of peptides with a high tendency to aggregate and form intermolecular β-sheet-like structures often leads to incomplete amino acid coupling, and therefore, poor overall yields.[^19b^^,^ ^20^] Strand E has previously been demonstrated to be highly aggregation-prone,[^16^] which makes it a particularly “difficult sequence” to synthesise.

Low yields and aggregation of the peptide sequence within the resin matrix were prevented by employing a low loading resin and inserting the β-sheet breaker 2-hydroxy-4-methoxybenzyl (Hmb) protecting group prior to the amyloidogenic sequence “FYLLYY” at position 12.[^21^] Use of a highly acid labile resin (NovaSyn TG Sieber resin) also allowed the fully deprotected peptide variants to be obtained by using a two-step cleavage procedure (Figure S1 in the Supporting Information). The peptides were first released from the solid support in their fully protected forms with a low concentration (2 % *v*/*v*) of trifluoroacetic acid (TFA). During this stage the aggregation-disrupting effect of the Hmb protecting group is retained, and prevents aggregation of the peptide variants within the matrix of the solid support. After release of the peptide from the support, global deprotection was achieved with a high concentration (94 % *v*/*v*) of TFA and appropriate cation scavengers. The series of peptides shown in Table 1 were finally purified by HPLC and shown to be the correct mass by ESI-MS.

**Table 1:** Peptides synthesised in this investigation.[a]

	Peptide target	Yield [%][b]
**control**	DWSFYLLYYTEFT	23
**p13T**	DWSFYLLYYTEF**pT**	10
**p10T**	DWSFYLLYY**pT**EFT	8
**p9Y**	DWSFYLLY**pY**TEFT	18
**p8Y**	DWSFYLL**pY**YTEFT	8
**p5Y**	DWSF**pY**LLYYTEFT	8
**p3S**	DW**pS**FYLLYYTEFT	12
**p3Sp10T**	DW**pS**FYLLYY**pT**EFT	9

[a] Phosphopeptide variants are labelled **pX**, where X denotes the phosphorylated amino acid side chain. All peptides were N-terminally acetylated and C-terminally amidated.

[b] Quoted yields are of pure peptide obtained after purification by preparative HPLC.

### Aggregation of nonphosphorylated strand E (control)

**Figure 1:**
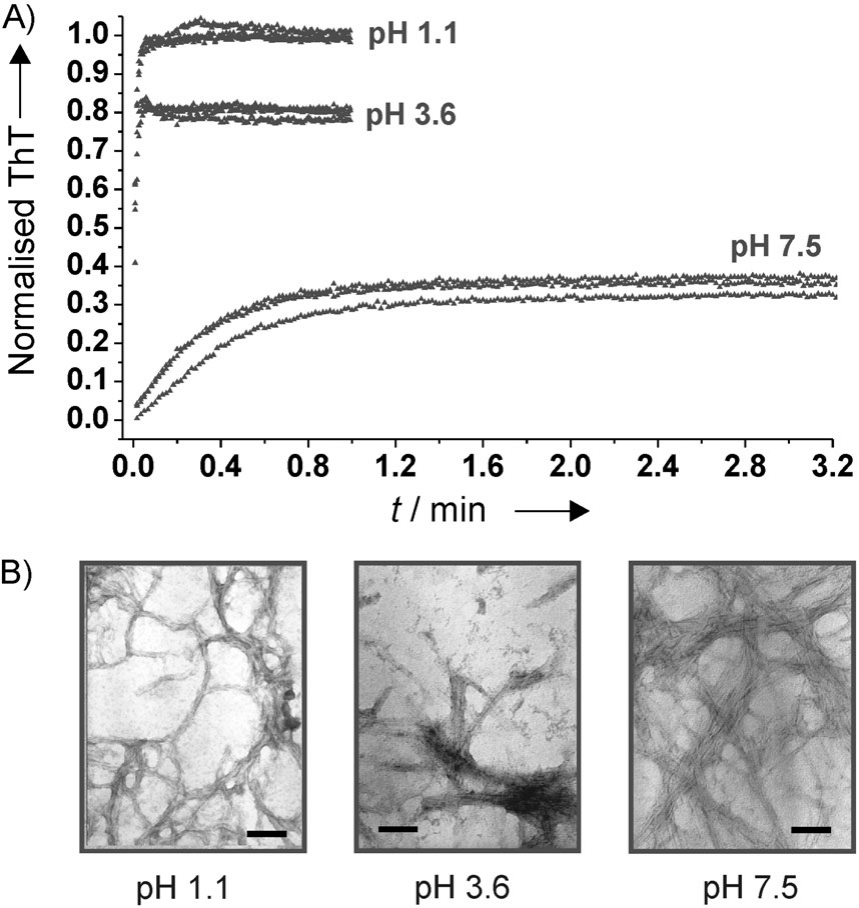
Aggregation of the non-phosphorylated peptide control at pH 1.1, 3.6 and 7.5 at 30 °C. A) Spontaneous fibril growth monitored by ThT fluorescence, and B) negative-stain TEM images of control after incubation for 4 days at the different pH values (scale bar: 100 nm). The kinetic curves shown in triplicate are representative of 40 replicates for each peptide obtained at each pH value. The ThT curves are normalised to the ThT fluorescence of samples at pH 1.1.

The aggregation kinetics of **control** were measured at pH 1.1, 3.6 and 7.5 by using ThT fluorescence and TEM as a probe of fibrillogenesis (Figure 1). These pH values were chosen to study the effect of three charge states of the phosphoamino acid moiety on the aggregation propensity of the peptides, ranging from 0, at acidic pH, to −2, at neutral pH. Aggregation of **control** was initiated by addition of a buffer solution containing ThT to a peptide stock solution in dimethylsulfoxide (DMSO) and the ThT fluorescence was measured in real-time in a 96-well plate format (Figure 1 A). The presence of fibrils was subsequently confirmed by TEM (Figure 1 B).

**Figure 2:**
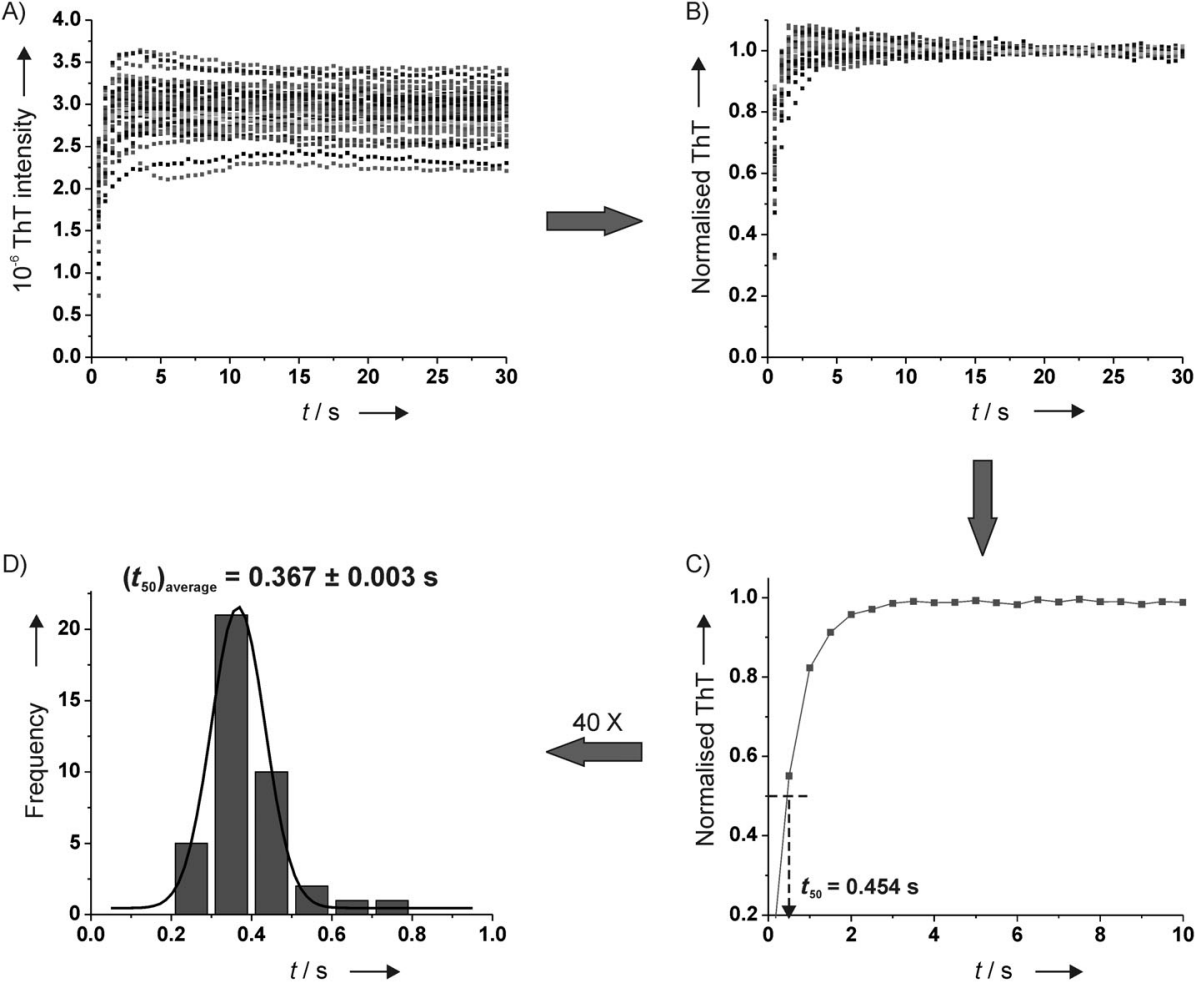
Process used to extract an average *t*_50_ value for control at pH 1.1. A) Raw data showing the 40 replicates after subtracting the DMSO blank, B) normalised data showing the 40 replicates after normalizing each curve against the average value of its plateau, C) extraction of a *t*_50_ value from a single normalised curve, and D) histogram of *t*_50_ values fitted with a Gaussian (black line) for all 40 replicate curves obtained. An average *t*_50_ value was calculated from the fit to the Gaussian function (Table S1 in the Supporting Information).

Analysis of the aggregation kinetics of **control** showed that the peptide formed fibrils rapidly at all pH values studied. The rate of fibril formation was then determined by using 40 replicate curves by extracting an average half-life value (*t*_50_) with the process described in Figure 2. A *t*_50_ value was extracted for each of the 40 normalised curves and these values were plotted on a histogram, which was then fitted to a Gaussian distribution to generate an average *t*_50_ value for each peptide under each set of conditions (Figure S2 and Table S1 in the Supporting Information).

The native sequence of strand E contains two negatively chargeable residues, aspartate and glutamate, at positions 1 and 11, respectively. Assuming p*K*_a_ values of approximately four for these groups, the net charge of **control** varies from 0 at pH 1.1 to −2 at pH 7.5, which correlates well with the increase of the *t*_50_ value between these pH values (0.367(±0.003) s at pH 1.1, 0.484(±0.006) s at 3.6, and 10.59(±0.18) s at pH 7.5). The presence of the two negative charges at the aspartate and glutamate residues thus retard significantly the rate of aggregation of the peptide, but are insufficient to inhibit fibril formation completely, even when fully charged at neutral pH (Figure 1 A and B).

### The effect of the phosphate group is electrostatic rather than steric

**Figure 3:**
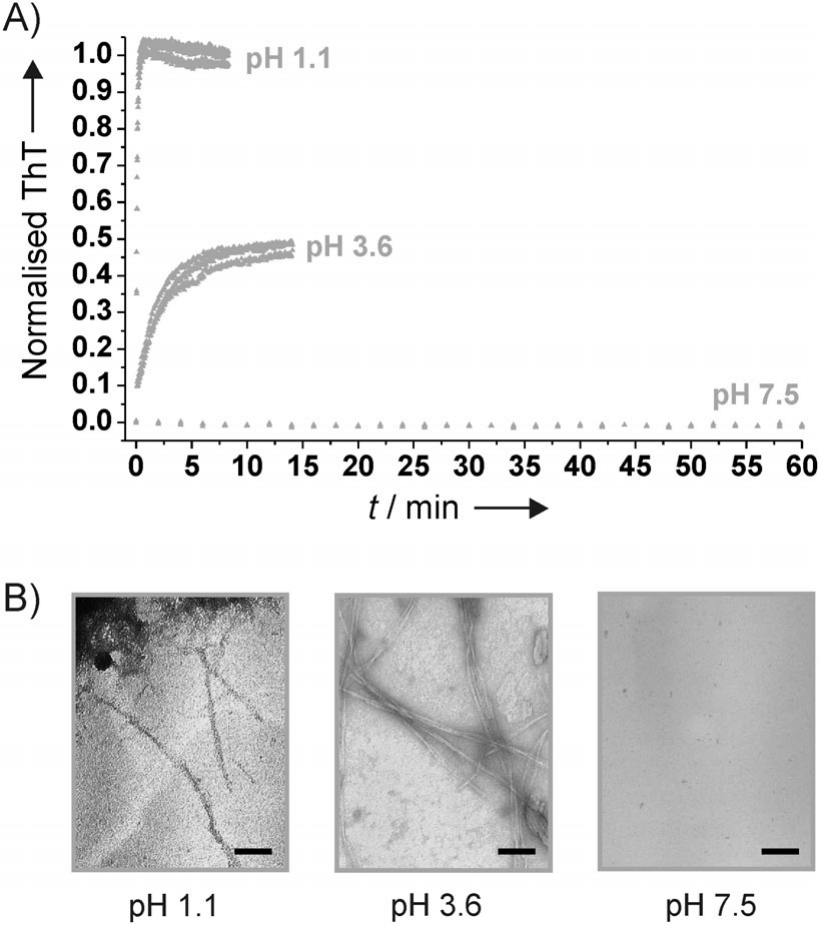
Fibril formation of the phosphopeptide variant p9Y at pH 1.1, 3.6 and 7.5 at 30 °C. A) Spontaneous fibril growth monitored by ThT fluorescence, and B) negative stain TEM images after incubation for 4 days (scale bar: 100 nm). The kinetic curves shown in triplicate are representative of 40 replicates obtained at each pH value.

To assess the effect of a single phosphate moiety on the aggregation kinetics of strand E, the phosphopeptide variant **p9Y** containing a single phosphotyrosine at position 9 was prepared and its aggregation kinetics were measured (Figure 3).

Whereas **control** formed fibrils rapidly at all pH values studied, the insertion of a phosphate group at position 9 into the sequence of strand E had a dramatic effect on assembly. While **p9Y** aggregated rapidly at pH 1.1 (*t*_50_=2.90(±0.02) s) and formed long straight fibrils as confirmed by TEM (Figure 3 B), the aggregation kinetics were retarded at pH 3.6 (*t*_50_=74.77(±0.77) s) and assembly was completely inhibited when the pH was increased to 7.5 (Figure 3 A and B). The ability of **p9Y** to form fibrils rapidly at pH 1.1 indicates that incorporation of a phosphate moiety at this site can be accommodated within the fibrillar architecture. Rather than being sterically determined, the inhibition of fibrillar assembly only occurs when the phosphotyrosine is in a negatively charged state.

### The position of the phosphate group is an important modulator of aggregation propensity

**Figure 4:**
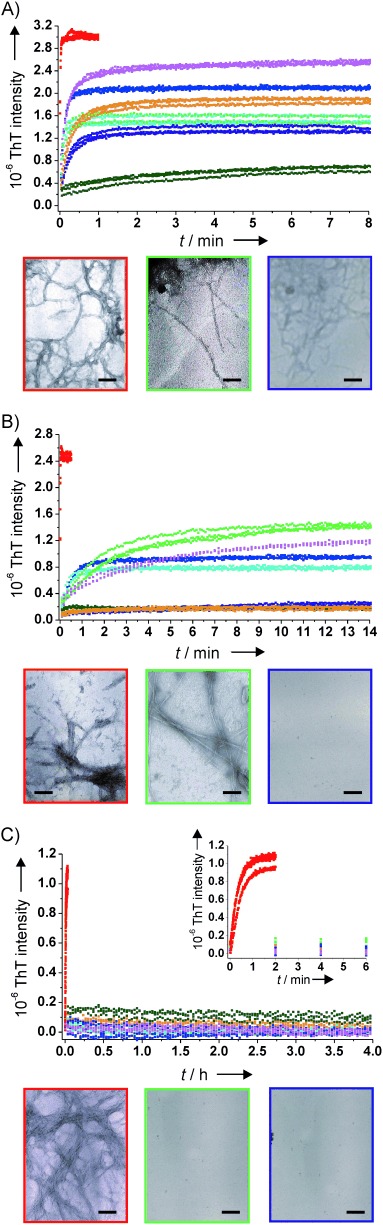
ThT kinetic curves of seven peptide variants of strand E at: A) pH 1.1, B) pH 3.6, and C) pH 7.5. Each peptide is coded by colour: control, p13T, p10T, p9Y, p8Y, p5Y, p3S and p3S10T. Representative negative-stain TEM images are shown after incubation of control, p9Y, and p5Y for 4 days at 30 °C (scale bar: 100 nm). Electron micrographs for each peptide at each pH value are shown in Figure S3 in the Supporting Information. The kinetic curves shown in triplicate are representative of 40 replicates for each peptide obtained at each pH value. An inset expansion is shown for pH 7.5, showing the first 6 min of assembly. Note the time axis in graphs (A), (B) and (C) are different.

**Figure 5:**
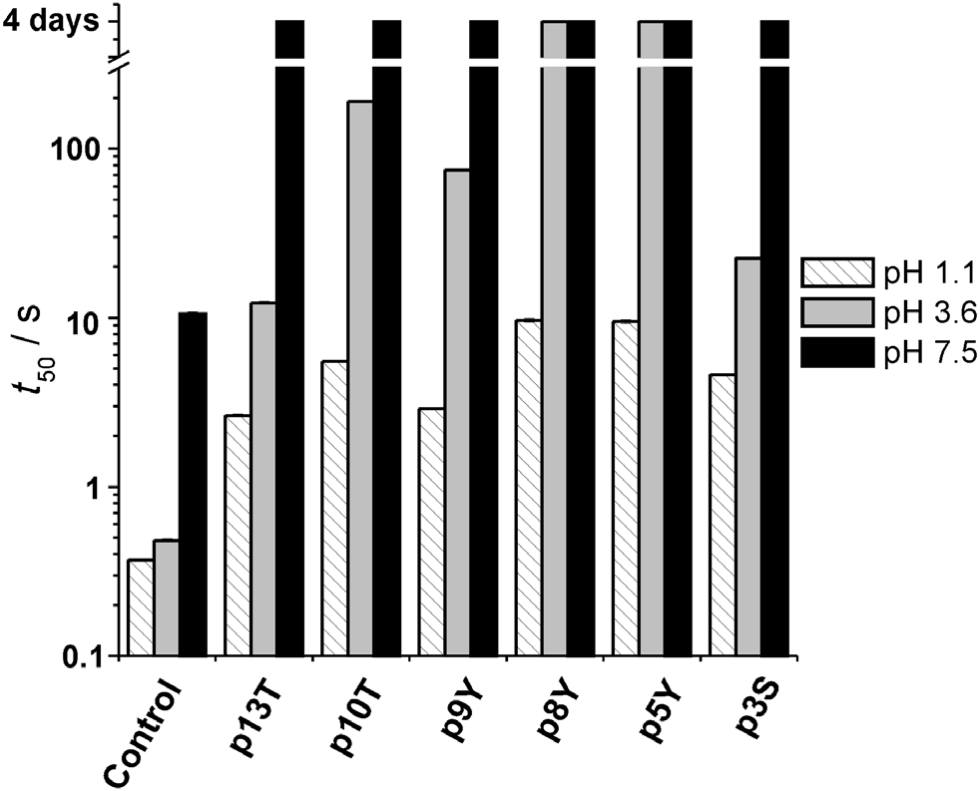
Histogram of average *t*_50_ values of aggregation of strand E peptides containing a single phosphorylated amino acid at pH 1.1, 3.6 and 7.5. Peptides showing no visible fibrils at the end of the incubation time (4 days) are shown with a *t*_50_ value of >4 days. The *t*_50_ values are plotted on a logarithmic scale. Standard deviations depicted as error bars are too small to be seen on this scale (Table S1 in the Supporting Information).

With these data in hand, we next investigated whether phosphorylation can be used as a general strategy for modulation of aggregation and whether the effect is sequence dependent. Accordingly, the phosphate group was introduced at seven different positions within the amino acid sequence of strand E (Table 1) and the aggregation kinetics of the different peptide variants were measured at pH 1.1, 3.6 and 7.5, as described for **control** (Figures 4 and 5). At pH 1.1, all the phosphopeptides aggregate rapidly to form amyloid-like fibrils, as measured by ThT fluorescence (Figure 4 A) and confirmed by negative stain TEM (Figure S3 in the Supporting Information). For all peptides containing a single phosphate group, the aggregation kinetics at pH 1.1 have *t*_50_ values below 10 s. For the variant containing two phosphate groups, **p3S10T**, the kinetic curves show a wide range of aggregation rates, ruling out determination of a single *t*_50_ value. Similar to the behaviour of **p9Y**, the aggregation kinetics of all other phosphopeptides was completely suppressed at pH 7.5 (Figure 4 C).

At pH 3.6, aggregation was highly dependent on the sequence, with each phosphopeptide variant exhibiting a different kinetic behaviour (Figure 4 B). Whereas four of the mono-phosphorylated peptides (**p13T**, **p10T**, **p9Y** and **p3S**) form fibrils at pH 3.6, two variants (**p5Y** and **p8Y**) were unable to form fibrils at this pH even after incubation for 4 days. Peptides **p3S** and **p10T** aggregated at pH 3.6 (Figure 5); however, the equivalent double phosphopeptide **p3S10T** did not form any fibrils under these conditions (data not shown).

### Inhibition of amyloid formation provides constraints on the fibril architecture

The sequence-dependent effect observed at pH 3.6 for the phosphopeptide variants described in Table 1 led us to consider whether the aggregation rates of the different peptides could provide information about the possible fibrillar architecture adopted by strand E. Assuming that electrostatic repulsion between adjacent phosphate moieties modulates fibril assembly, and that the different peptide sequences assemble into a common fibril morphology, as is frequently (but not always) found for close sequence homologues,[^22^] the different assembly properties of the peptides at pH 3.6 (under which conditions their assembly rates are most different) can be exploited to derive models of the fibril architectures most likely to be formed.

**Figure 6:**
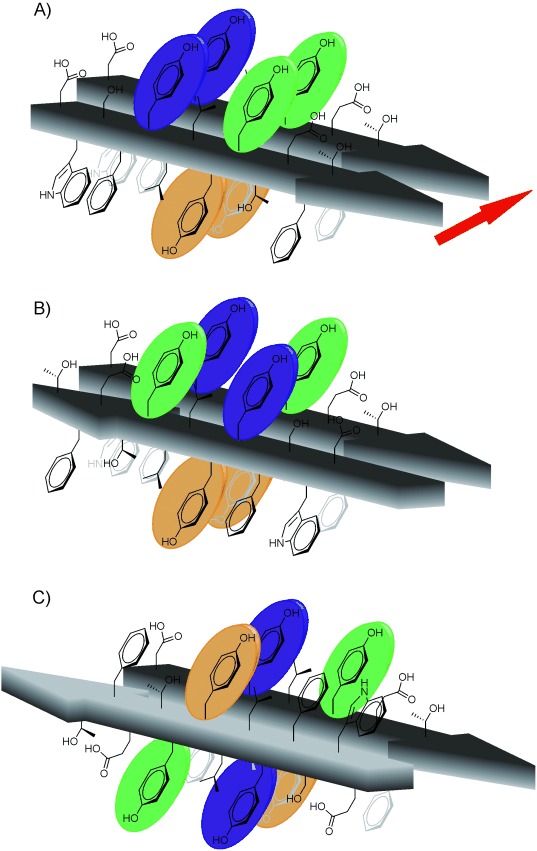
Schematic representation of the possible strand–strand interactions within a β-sheet for strand E with the β-strands: A) parallel, in-register, B) antiparallel, in-register, and C) antiparallel, shifted arrangement. The coloured amino acids represent the tyrosine residue in which the phosphate groups would be present: p9Y (green), p8Y (orange) and p5Y (purple). The red arrow shows the long axis of the fibrillar array.

Eisenberg et al. have defined a limiting set of eight possible arrangements of peptides in a cross-β structure. These structures are referred to as “steric zippers” because the side chains of residues in adjacent β-sheets intermesh tightly like the teeth of a zipper to form a dry interface.[^12^] These structures are formally defined by the symmetry relationships in the organisation of the β-strands and β-sheets with respect to one another. We first considered the first level of the steric zipper architecture—the arrangement of β-strands within a β-sheet—to determine whether the fibrillation kinetic data for different phosphovariants of strand E could support, or eliminate, certain classes. The most simplistic representation of a steric zipper consists of a pair of β-strands that can be organised in one of three arrangements. While only one parallel arrangement is possible, a β-strand can generate two antiparallel sheet structures, depending on whether the β-strands are related by twofold rotational (*C*_2_) symmetry around the backbone axis or perpendicular to the backbone axis. The alternative operations modulate the orientation of side chains that project from the β-strands and also lead to a shifted or in-register organisation of the β-strands (Figure 6).[^23^]

Three simplified strand–strand schematic models of **control** were constructed in these three strand architectures with the tyrosine residues coloured according to the position of the phosphate groups in **p5Y**, **p8Y** and **p9Y** (Figure 6). These peptide variants were chosen because they show very different rates of aggregation at pH 3.6, with both **p5Y** and **p8Y** (orange and purple) being incapable of forming fibrils at this pH, while **p9Y** (green) aggregates rapidly.

Assuming that modulation of the aggregation kinetics of the different phosphopeptides results from electrostatic repulsion between adjacent phosphate groups, the proximity of the phosphotyrosine residues was used to determine whether peptide E is most likely to adopt parallel or antiparallel β-strands in the fibril structure. Peptides **p5Y** and **p8Y** do not aggregate at pH 3.6, whereas **p9Y** is able to form fibrils at this pH. Using these criteria and experimental observations, a parallel, in-register organisation of β-strands (Figure 6 A) can be ruled out since in this arrangement of β-strands each phosphorylated amino acid is positioned equivalently within the β-sheet. Similarly, an antiparallel, in-register arrangement of strands within the β-sheet (Figure 6 B) cannot account for the different aggregation properties of peptides **p5Y** and **p9Y** as the tyrosine residues at positions 5 and 9 are equally spaced. The only β-sheet arrangement that can account for the different behaviours experimentally observed for **p5Y**, **p8Y** and **p9Y**, therefore, is the antiparallel shifted organisation within a β-sheet (Figure 6 C). This model accounts fully for the similar aggregation kinetics of **p5Y** and **p8Y** since the phosphotyrosine groups are spaced identically in this β-sheet structure. By contrast, the separation of the tyrosine residues bearing the phosphate group in **p9Y** is greater in this fibril structure accounting for the ability of **p9Y** to form fibrils at pH 3.6. This structure also allows the maximum π–π stacking interactions in the core sequence, and the alignment of the hydrophobic leucine residues—features presumed to play an important role in determining fibril stability.[^24^]

**Figure 7:**
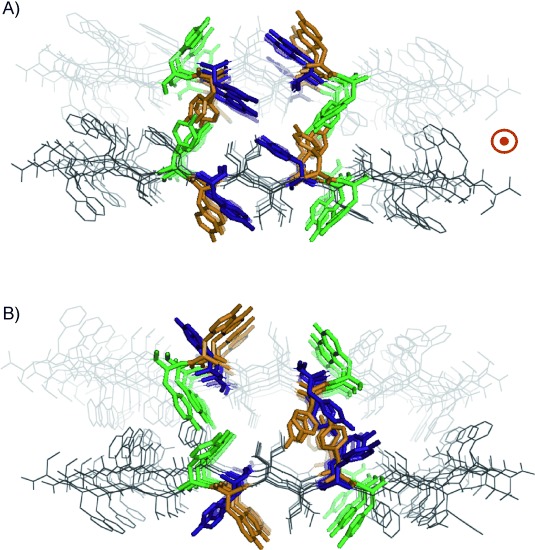
The 8×2 steric zippers of strand E control showing the intersheet packing for: A) class 7, and B) class 8, as described by Eisenberg et al.^12^ The coloured amino acids represent the tyrosine residues that are phosphorylated in p9Y (green), p8Y (orange) and p5Y (purple). The long axis of the fibrillar array is shown as a red dot, the fibrillar axis goes into the plane of the paper.

Of the eight classes of steric zippers described by Eisenberg et al., only two classes possess the β-strand symmetry of the shifted organisation described in Figure 6 C: the class 7 or 8 architectures.[^23^] We, therefore, considered if the data presented above can allow discrimination between the different β-sheet packing modes within the fibril architectures described by these alternative classes. While the arrangement of a pair of β-strands can allow certain models to be ruled in or out as discussed above, these simplistic strand–strand schematics do not account for the contacts occurring at the β-sheet interface. Models of aggregates equivalent to a pair of interdigitating β-sheets of eight β-strands were therefore built in silico for fibrils with class 7 and 8 architectures with the nonphosphorylated peptide (**control**; Figure 7), assuming an antiparallel shifted β-strand organisation within each β-sheet. The difference between these two steric zippers relates to the relative orientation of the faces of the two identical β-sheets. In order to determine which class of steric zippers is the more likely arrangement for strand E fibrils, an average distance between the C_β_ atoms of the tyrosine residues at positions 5, 8 and 9 was calculated (Table 2).

**Table 2:** Average C_β_–C_β_ distance between tyrosine residues at positions 5, 8 and 9 in a steric zipper of class 7 and 8.

Peptide	Class 7 C_β_–C_β_ [Å]	Class 8 C_β_–C_β_ [Å]
**5Y**	13.7	11.6
**8Y**	13.6	10.4
**9Y**	20.5	10.6

The experimental results obtained at pH 3.6 show that peptides **p5Y** and **p8Y** are unable to form fibrils (Figure 5), while **p9Y** aggregates rapidly at this pH; this suggests that the phosphotyrosine groups of **p5Y** and **p8Y** must be in closer proximity than that of **p9Y** in the intersheet packing in the fibril structure. In a class 8 architecture (Figure 7 B), the average C_β_–C_β_ distance between the tyrosine residues of **5Y**, **8Y** and **9Y** (corresponding to the position of the three tyrosine residues where the phosphate groups have been introduced) is comparable (Table 2). By contrast, in a class 7 architecture, **5Y** and **8Y** have comparable C_β_–C_β_ distances, while the tyrosine residues of **p9Y** are significantly further apart. The class 7 architecture is therefore more consistent with the aggregation propensities of the peptides phosphorylated at these residues. Analysis of these two models thus suggests that a steric zipper with a class 7 architecture is the only organisation consistent with the experimental results obtained (Figure 7 A).[^12^]

### A class 7 architecture as a viable polymorph of strand E

**Figure 8:**
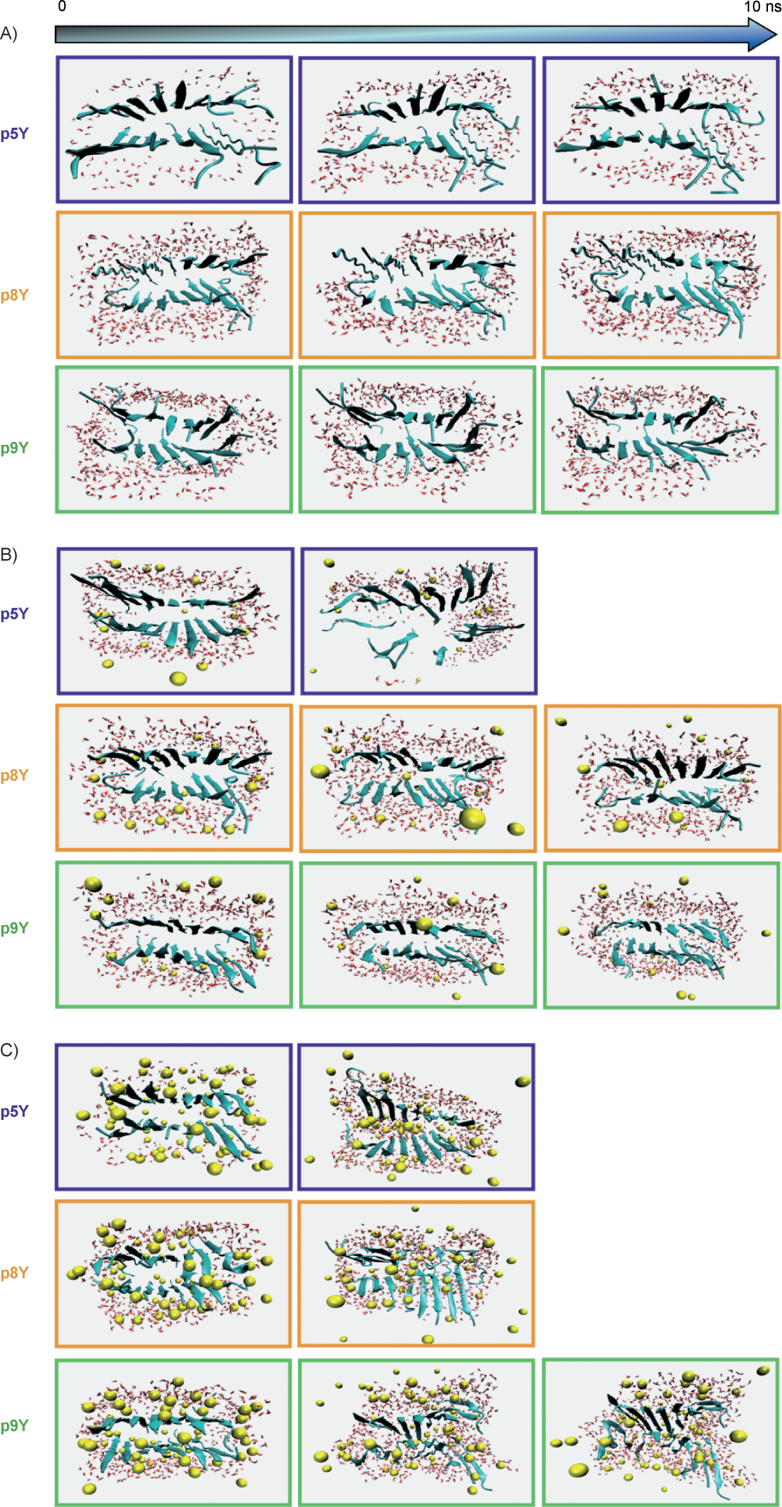
Snapshots of the MD simulations carried out in explicit solvent for the phosphopeptide variants p5Y, p8Y and p9Y bearing: A) a fully protonated, B) a mono-anionic, and C) a di-anionic phosphate group. Snapshots taken at *t*=0, 5 and 10 ns were prepared with VMD.^[25]^ The water molecules (red dots) are represented as a surface area within 10 Å of residues Leu and the phosphotyrosine, and the yellow spheres represent the sodium ions used to neutralise the overall structure.

If a polymorph corresponding to a class 7 architecture is preferentially formed across the peptides and pH range studied, the stability of the models built for the phosphopeptide variants **p5Y**, **p8Y** and **p9Y** with a class 7 architecture should correlate directly with the aggregation behaviours observed experimentally. To determine whether this is the case and to provide a deeper insight into the inter-atomic interactions within fibrils containing phosphotyrosine moieties at different sites within a common class 7 architecture, in silico models of fibrils containing eight β-strands and two β-sheets with charged and protonated phosphotyrosine residue at positions 5, 8 or 9 were built. Using atomistic MD simulation, we then monitored whether the fibril remained as a stacked pair of ordered β-sheets as would be expected for stable architectures. The simulations are used as a design tool based on the assertion that if the electrostatic repulsion between charged phosphate moieties within the fibril is sufficiently large to prevent peptide aggregation experimentally, the originally ordered fibrils built in silico will collapse into a disordered state during the MD calculations. MD trajectories 10 ns in length in explicit solvent were therefore obtained for the structures bearing either fully protonated, mono- or di-anionic phosphotyrosine groups at each site. Figure 8 shows snapshots of the structures at 0, 5 and 10 ns of the MD simulations in which the solvent distribution around the leucine and the phosphotyrosine residues of **p5Y**, **p8Y** and **p9Y** are highlighted. The results are striking, and suggest that the solvent and counter-ion environment plays an important role in determining the behaviour of the fibrils decorated with phosphotyrosine groups carrying differing amounts of charge, as discussed in detail below.

With a fully protonated phosphotyrosine group, which is electrostatically neutral (Figure 8 A), the steric zippers of **p5Y**, **p8Y** and **p9Y** showed little or no variation in their architectures throughout the simulation, and the dry, intersheet interface remained solvent-free over the duration of the MD. As no water molecules penetrated the interface the side chains of both β-sheets remained dry and tightly packed. These results suggest that the phosphopeptide variants **p5Y**, **p8Y** and **p9Y** bearing a fully protonated phosphotyrosine group can adopt a class 7 architecture and accommodate a phosphate group inside the tight dry interface while remaining in an ordered fibrillar state. Transient hydrogen bonding interactions between phospho groups within the interface could be observed in the structure of **p5Y** and to a lesser extent in the structure of **p8Y.** In addition, the phophotyrosine group in **p8Y** and **p9Y** zippers showed transient intersheet hydrogen bonding to the hydroxyl group of Y9 and Y8, respectively, during the simulations. The MD simulations are thus consistent with the aggregation behaviour observed experimentally for **p5Y**, **p8Y** and **p9Y** at pH 1.1.

By replacing the fully protonated phosphotyrosine group with its di-anionic form (Figure 8 C), thereby introducing a −2 electrostatic charge per phosphotyrosine group, the stability of the steric zippers of **p5Y**, **p8Y** and **p9Y** was greatly affected. During the simulation, water molecules and sodium counter-ions penetrated the dry interface, leading to the dissociation of each steric zipper. The phosphopeptide variants **p5Y**, **p8Y** and **p9Y** bearing a di-anionic phosphate group are therefore energetically unstable, such that electrostatic repulsion and/or preferential solvation of the negatively charged phosphotyrosine groups results in disassembly of these structures at neutral pH. These results correlate with the inhibition of the aggregation of **p5Y**, **p8Y** and **p9Y** observed by ThT fluorescence at pH 7.5.

**Figure 9:**
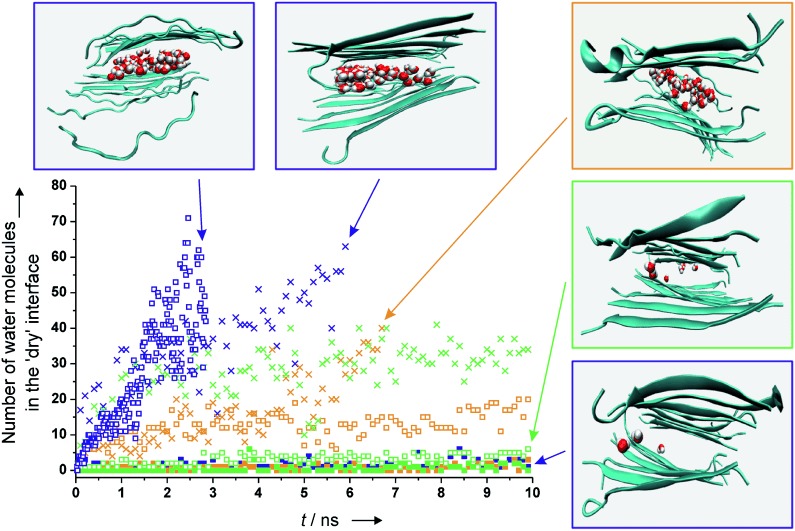
Number of water molecules found within the β-sheet interface of the class 7 steric zipper of p5Y, p8Y and p9Y over the duration of the MD simulations. Snapshots highlighting the position of the water molecules at the end of the simulations are shown for some fibrillar models (snapshots of all three peptides with the three different protonation states are summarised in Figure S4 in the Supporting Information). The phosphopeptide variants are coloured accordingly to the position of their phosphate groups, p9Y (green), p8Y (orange) and p5Y (purple). The different protonation states of the phosphate group are represented as follow: 0 with solid squares, −1 with open squares, and −2 with crosses.

Similar to the results obtained experimentally, when the phosphotyrosine group was replaced with its mono-anionic form a sequence-dependent effect was also observed by MD simulations (Figure 8 B). While the steric zippers of **p5Y** and **p8Y** rapidly disassemble as water molecules and sodium counter-ions penetrate, the dry interface formed by the two stacked β-sheets, the steric zipper of **p9Y** remained free of water molecules and sodium counter-ions throughout the simulation. As previously shown in Table 2, the phosphotyrosine groups of **p9Y** are much further apart in the intersheet packing than those of **p5Y** and **p8Y**, with the consequent reduction in electrostatic repulsion within the steric zipper of **p9Y** in this fibril structure. Considering that the interface formed by the tight packing of the side chains must be free of solvent or counter-ions for a steric zipper to be considered stable, the structural integrity of the fibrillar models built for **p5Y**, **p8Y** and **p9Y** can be quantitatively assessed by calculating the number of water molecules penetrating the dry interface over the duration of the trajectories (Figure 9).

These data show that the models generated for **p5Y**, **p8Y** and **p9Y** bearing protonated phosphotyrosine groups remained dry with less than five molecules of water penetrating within the β-sheet interface over the duration of the MD simulations. When the phosphotyrosine groups were replaced with their mono-anionic equivalent, the water penetration appeared to be directly related to the position of the phosphotyrosine group in the sequence of strand E. While less than five water molecules penetrate the β-sheet interface of **p9Y**, the structures of **p5Y** and **p8Y** are rapidly dissolved following the diffusion of a large number of water molecules (ca. 60 and 20 water molecules, respectively) within the interface (Figure 9). The dissolution effect observed in the MD simulations with a mono-anionic phosphotyrosine group is therefore sequence dependent and correlates precisely with the aggregation behaviour observed experimentally at pH 3.6 (Figures 4 B and 5). With a di-anionic phosphate group, the simulations show a rapid solubilisation of the fibrillar models of **p5Y**, **p8Y** and **p9Y** (ca. 55, 35 and 35 waters molecules penetrating the dry interface, respectively) independent of the position of the phosphate group in the sequence of strand E (Figure 9). Taken together, therefore, the MD simulations and experimental data of variants **p5Y**, **p8Y** and **p9Y** obtained at pH 1.1, 3.6 and 7.5 imply that strand E adopts a class 7 fibrillar architecture that is uniquely able to accommodate a singly charged phosphotyrosine within its dry β-sheet interface.

## Conclusion

The ability of post-translational modifications to modulate the aggregation propensity of peptides and proteins in vivo is important in the progression of a range of amyloid related diseases.[^14^^,^^25^] In this study we have significantly advanced the understanding of how modulation of amyloid formation by post-translational modifications can be understood at the atomistic level. By combining experimental and theoretical studies of a model peptide we have revealed new features of how phosphorylation can modulate the aggregation of a peptide sequence. Examining the influence of both position and charge of the modified residues on the aggregation rates reveals that phosphorylated residues can be sterically accommodated within the steric zippers of cross-β assemblies, but that as the residues become highly charged aggregation is strongly inhibited. At intermediate charge states the effect of phosphorylation becomes sequence dependent. The sequence-specific effects have enabled us to provide predictive insights into the possible architecture of the fibrils. By combining the experimental data with atomistic MD simulation of possible polymorphs we gain a consistent picture in which experimental aggregation propensity and measure of fibril stability in silico suggest a class 7 steric zipper for the fibril architecture of this peptide sequence.

The results presented show that phosphorylation provides a powerful and tunable control of aggregation propensity that can be exploited as a switch able to turn on/off the aggregation of self-assembling materials. Accordingly, assembly can be triggered by simple pH switch, or by removal of the phosphate group.[^14^^,^^15^] Indeed, dephosphorylation of **p9Y** was efficiently performed in the presence of alkaline phosphatase at pH 8.9 to generate dephosphorylated peptide E, which aggregated with a similar *t*_50_ to the structurally identical, synthetic equivalent (data not shown). One of the most fascinating features of amyloid is the apparent diversity of structures available to a single polypeptide sequence within a generic cross-β architecture.[^12^^,^^26^] The ability to identify which sequence can assemble into which polymorph is a major challenge that is crucial for the development of molecular understanding of amyloid disease and to exploit amyloid as a functional nanomaterial. In addition to its practical utility, the data presented suggest that phosphoamino acid scanning might be a useful method for the establishment of putative fibrillar architectures and polymorph distinction, which can then inform the design of methods of structural confirmation. Currently, generation of such atomistic descriptions of amyloid is a major undertaking, typically involving MAS-NMR spectroscopy and cryo-EM. High resolution structures of amyloid that are consistent with both long and short range information from such experiments, however, have been achieved for very few systems to date.[^27^] In this study we have shown that simple chemical modification in combination with simulation can be used to develop fibril models efficiently and rapidly and will be of wide utility in providing rapid development of structural models for other assemblies of other peptides. The simulations also suggest mechanisms by which the change in charge state of the phosphorylated residue modulates aggregation. Rather than electrostatic repulsions simply forcing aggregates apart, charge density in the interior of the steric zipper promotes the invasion of ions and water into the intersheet space promoting dissolution of the assembled architecture.

## Experimental Section

**Peptide synthesis:** Peptide variants were synthesised manually by using standard solid-phase techniques for Fmoc *N*α-protected amino acids on a NovaSyn TG Sieber resin (0.20 mmol g^−1^, Novabiochem).[^19b^] Standard Fmoc-protected and phosphorylated amino acids (*N*-α-Fmoc-*O*-benzyl-l-phospho-serine, -threonine and -tyrosine, Novabiochem) couplings were carried out with excess 2-(6-chloro-1*H*-benzo-triazole-1-yl)-1,1,3,3-tetramethylammonium hexafluorophosphate (HCTU, Novabiochem) and *N*,*N*-diisopropylethylamine (DIPEA, Aldrich) in *N*,*N*-dimethylformamide (DMF). The β-sheet breaker *N*-α-Fmoc-*N*-α-(2-Fmoc-oxy-4-methoxybenzyl)-l-Phe (Fmoc-(FmocHmb)Phe-OH, Novabiochem) was inserted at position 12 to prevent on-resin aggregation by using HCTU and DIPEA, and the subsequent amino acid was coupled, overnight, with excess 2-(7-aza-1*H*-benzotriazole-1-yl)-1,1,3,3-tetramethylammonium hexafluorophosphate (HATU, Novabiochem) and DIPEA. Deprotection of the Fmoc-protecting group was achieved with a solution of piperidine (20 %) in DMF. Couplings and deprotections were confirmed by using the Kaiser test.

Acetylation of the N terminus was achieved with excess acetic anhydride and DIPEA in DMF. Cleavage and side-chain deprotection was accomplished in two steps. The peptide was first released from the resin with a solution of DCM/TFA (98:2) and the resulting solid was finally deprotected in solution with a cleavage cocktail of TFA/EDT/H_2_O/PhOH (94:2:2:2), precipitated in cold diethyl ether and lyophilised from AcOH (0.1 %).

The crude peptides were purified by preparative reverse phase HPLC on a Gilson instrument with a Phenomenex Jupiter Proteo column (10 μm, 90 Å, 250×21.2 mm, 20 mL min^−1^) and eluted with a linear gradient of 5 % to 25 % B, where B is acetonitrile+0.1 % ammonia and A is water+10 % B. Analytical traces and accurate mass data were obtained to confirm purity.

**Measurement of aggregation by using ThT fluorescence:** All fluorescence experiments were performed at 30 °C on a Perkin–Elmer EnVision 2103 Multilabel reader (excitation filter: photometric 440, band width: 8 nm; emission filter: FITC 485, band width: 14 nm; general beam splitter in the optical path; ten flashes per well), by using a 96-well plate (MicroFluor). Buffers with a final ionic strength of 150 mM (A: 25 mM glycine, 47.0 mM NaCl (pH 1.1); B) 25 mM sodium phosphate, 25 mM sodium acetate, 99.7 mM NaCl (pH 3.6); and C) 25 mM sodium phosphate, 83.5 mM NaCl (pH 7.5)) were mixed in a 9:1 ratio with ThT (1 mM in water). Peptide stock solutions (2 μL, 1 mM in DMSO) were diluted to a final concentration of 20 μM with ThT buffer (98 μL). In the negative control the peptide was replaced with DMSO.

At pH 1.1 and 3.6, buffers A and B, respectively, were dispensed directly from the plate reader into a 96-well plate, containing either the peptide stock solution or DMSO. At pH 7.5, buffer C was dispensed with a Hamilton Star robotic liquid handler into a 96-well plate and the plate was sealed with the optically clear film Platemax (Axygen). A continuous assay was used to monitor fibril formation (at pH 1.1, 250 measurements at intervals of 2 s; at pH 3.6, 170 measurements at intervals of 5 s apart for **p10T** (10 s intervals); at pH 7.5, 100 measurements at intervals of 4 min).

The fluorescence intensity of each sample was corrected for the ThT signal of the negative control (Figure S2 in the Supporting Information). Control experiments with preformed fibrils (incubated in neat buffer at a final concentration of 40 μM for 4 h) demonstrated that ThT binding to the preformed fibrils occurs within the dead time of less than 0.5 s at all pH values studied.

**The** ***t***_**50**_ **determination:** The 40 kinetic curves were each normalised to the average of their end point (plateau), and because they could not be analytically fitted, the time to reach 50 % of the maximum signal (*t*_50_) was numerically calculated for each curve by using Equation (1):

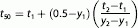
(1)

where *t*_1_ represents the time point below *t*_50_ and *t*_2_ the time point above; *y*_1_ and *y*_2_ are the normalised fluorescence intensities at *t*_1_ and *t*_2_, respectively.

For each peptide at each pH, a frequency count was carried out for the 40 *t*_50_ values obtained with a total number of bins equal to 10 and a histogram was plotted. A Gaussian curve was fitted to each histogram, and mean *t*_50_ values and standard deviations were calculated for each peptide for each set of conditions based on a normal distribution.

**Transmission electron microscopy (TEM):** Samples were prepared in an Eppendorf tube by diluting peptide stock (10 μL; 1 mM in DMSO) in buffer (500 μL). After incubation for 4 days at 30 °C with shaking at 200 rpm, the solution (10 μL) was dispensed undiluted on to freshly ionised formvar- and carbon-coated TEM grids (Agar) and incubated for 1 min. The grids were stained for 30 s with uranyl acetate (4 % *w*/*v*). All images were taken by using a Philips CM10 electron microscope operating at 80 keV.

**Model minimisations:** Energy minimisation was carried out by using the AMBER 9 suite of programs in conjunction with the AMBER 99 forcefield.[^28^] The non-phosphorylated peptide, DWSFYLLYYTEFT, containing an acetyl N terminus and a NH_2_ C terminus, was first built as a monomeric strand; the steric zippers corresponding to class 7 and 8 were then constructed as two sheets of eight β-strands each, separated by 12 and 4.8 Å, respectively, by using the NAB molecular building tool to generate two antiparallel, shifted fibril models.[^29^] Side-chain C_α_ dihedral angle values *χ*_1_ were altered by using the Richardson rotamer library.[^30^] The minimisations were carried out in implicit solvent with the generalised Born/Surface Area (GB/SA) method,[^31^] by using the Tsui and Case parameters,[^32^] and an interaction cut-off of 25 Å. The energy minimisation used steepest descent for ten cycles, followed by a switch to conjugate gradient for the remaining 9990 cycles. Molecular representations showing structures at the end of the minimisation of the structures were produced by using PyMOL.[^33^]

**MD simulations:** Trajectories were first obtained by using the GB/SA implicit solvent model, as the absence of solvent friction from discrete water molecules will accelerate the in silico collapse of an ordered array if repulsive interactions outweigh favourable interactions between the peptide strands.

The class 7 models obtained from the GB/SA minimisations were pre-equilibrated for 80 ps with coordinate, intersheet and hydrogen bond restraints, then 1 ns without the coordinate restraints, and finally between 10 and 20 ns trajectories were obtained by running the structures unrestrained. A PDB file of the last snapshot was made and fully protonated phosphotyrosine residues (Y0P) were introduced at positions 5, 8 and 9 by using Amber parameters.[^34^] These new structures were minimised, pre-equilibrated with restraints and run for 10 ns in GB/SA unrestrained. A PDB file of each structure obtained was made. The PDB files of the Y0P fibrils were solvated by using a TIP3P solvation box,[^35^] minimised, pre-equilibrated and run unrestrained for 10 ns.

Fibrillar structures containing a mono- or di-anionic phosphate group (Y1P and Y2P, respectively) were obtained by replacing Y0P in the PDB files from the GB/SA trajectories with either Y1P or Y2P.[^34^] The structures generated were solvated with a TIP3P solvation box,^35^ neutralised with counter-ions (Na^+^), minimised in two steps, pre-equilibrated with coordinate restraints and finally run unrestrained for 10 ns.
