# Supplementary material for: Phosphorylation as a Tool To Modulate Aggregation Propensity and To Predict Fibril Architecture
Source: Chembiochem. 2011 Dec 15;13(2):271–81. doi: 10.1002/cbic.201100607 (PMC3531611; doi:10.1002/cbic.201100607)
Supplement: Supplementary file 1 [file cbi00013-0271-sd1.pdf]

Supplementary Information

1. Synthesis of Peptides

Materials.

All reagents were obtained from commercial sources and used without further purification: DIPEA (N,N-diisopropylethylamine) from Alfa Aesar, calf intestinal alkaline phosphatase (CIP) from NewEngland Biolabs. DMF (N,N-dimethylformamide, HPLC grade), EDT (ethanedithiol) and piperidine were acquired from Fluka. ThT (thioflavin T), diethyl ether and acetic anhydride were purchased from Sigma-Aldrich. DCM (dichloromethane, HPLC grade), acetonitrile (HPLC grade) and ammonia were purchased from Fisher Scientific. HCTU (2-(6-chloro-1H-benzotriazole-1-yl)-1,1,3,3-tetramethylaminium hexafluorophosphate), HATU (*2*-(7-azabenzotriazol-1-yl)-1,1,3,3-tetramethyluronium hexafluorophosphate), Fmoc-amino acids and NovaSyn® TG Sieber resin were purchased from Novabiochem. TFA (trifluoroacetic acid) was acquired from Sigma-Aldrich, was freshly distilled and used within 8 weeks. Water was purified using a Millipore MilliQ water purification system.

HPLC.

Analytical HPLC were carried out on a Dionex HPLC system using a Chromeleon® interface. Analyses were monitored at both 210 and 278 nm. Separations were performed using a Phenomenex Jupiter® Proteo column (4 μm, 90 Å, 250x4.6 mm) at a flow rate of 1 mL/min. The samples were eluted with variable gradients of A: 0.1% ammonia in water and B: 10% A in CH_3CN.

Semi-preparative HPLC was performed on a Dionex HPLC system equipped with a Phenomenex Jupiter® Proteo column (10 μm, 90 Å, 250x10.0 mm) at a flow rate of 5 mL/min. The UV detection was set at both 210 and 278 nm.

Preparative HPLC was performed on a Gilson HPLC system equipped with a Phenomenex Jupiter® Proteo column (10 μm, 90 Å, 250x21.2 mm) at a flow rate of 20 mL/min. The UV detection was set at 278 nm.

Mass spectrometry.

Nominal mass spectrometry was performed on a Waters/Micromass ZMD or a Bruker HCTUltra instrument, using electrospray ionisation (ESI). Accurate mass data were obtained on a Bruker MicrOTOF instrument also using electrospray ionisation.

Peptide Synthesis

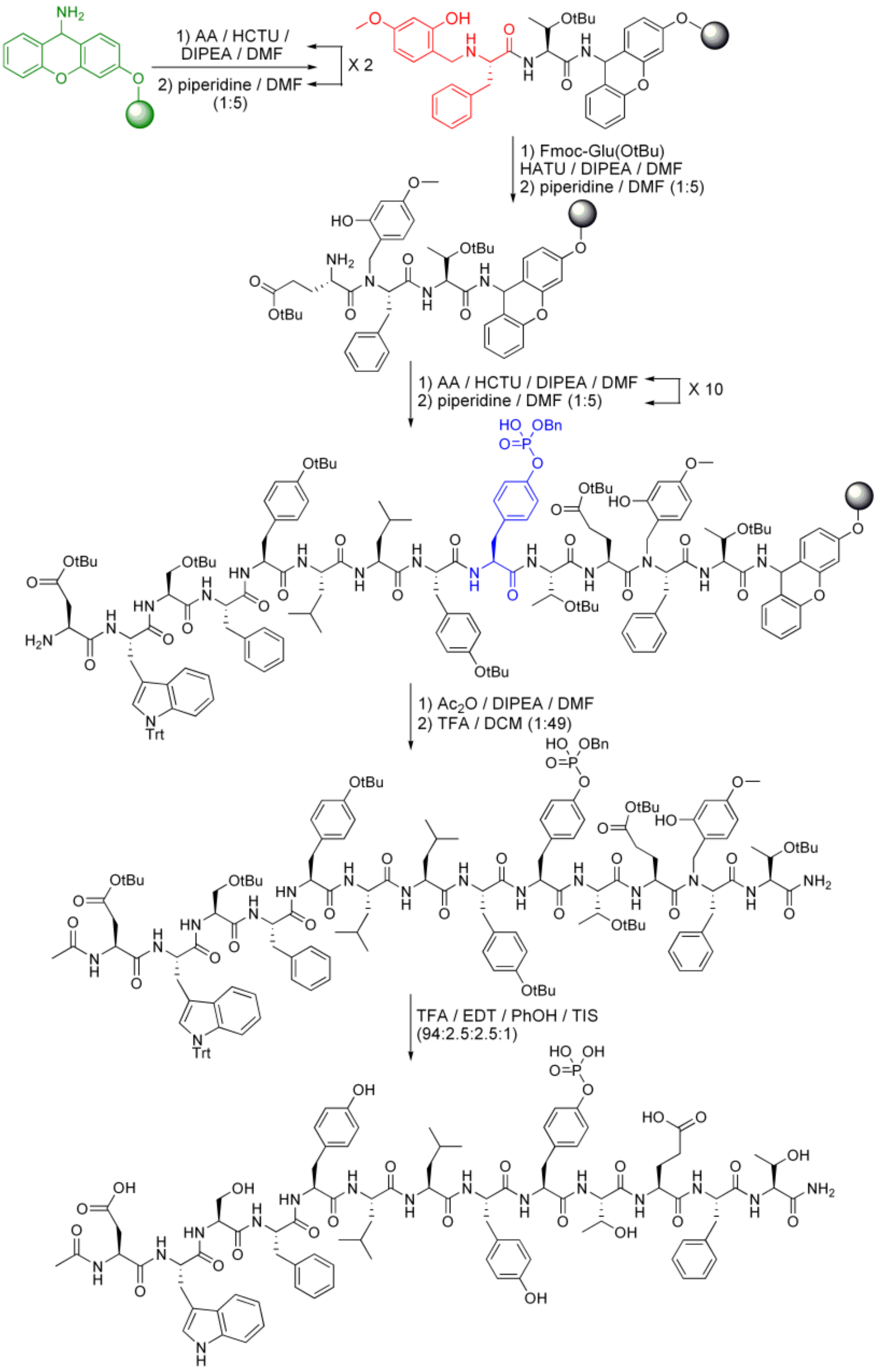

Figure S1. Example synthesis of the phosphopeptide variants of strand E **p9Y**. The peptidic chain was prepared on a low loading, highly acid labile Sieber amide resin (green) with the inclusion of a β-sheet breaker (Hmb-protected Phe, shown in red) and mono-benzyl phosphorylated amino acids (blue).

Analytical data

Compound ***1*** (Ac-DWSFYLLYYTEFT-NH_2):

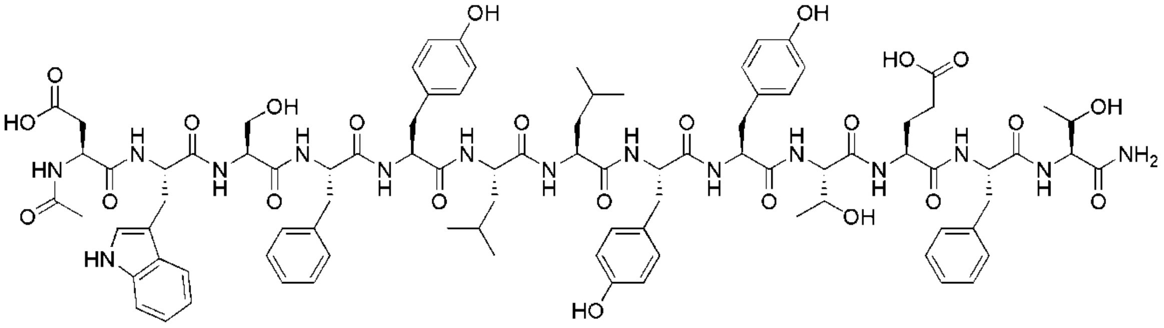

RP-HPLC (Phenomenex Jupiter® 4 μm Proteo 90Å 250 x 4.6 mm): flow rate 1 mL/min; gradient elution t=0, A:B 95:5 ; t=30 1:1 ; t=32 0:100 ; Rt 20.3 min (99% pure).

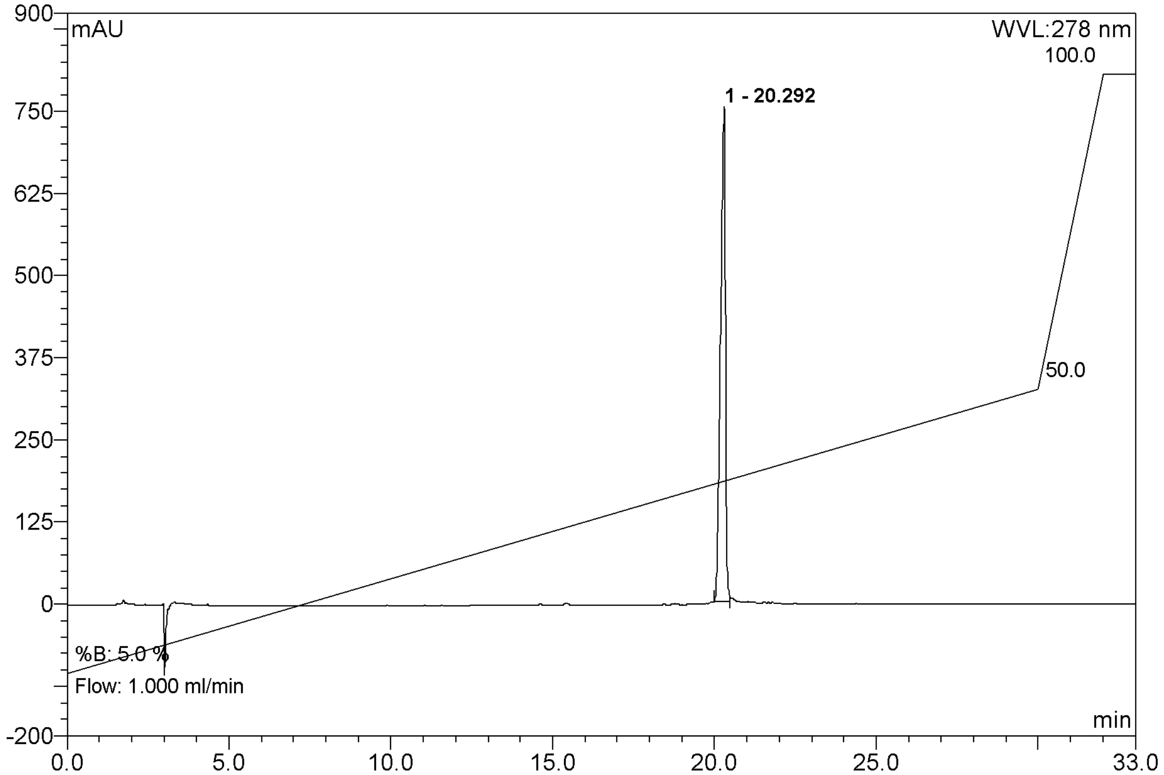

MS (ESI) m/z calcd for $C_{90}H_{111}N_{15}O_{24}$ $[M-2H]^{2-}$: 892.8969, found: 892.9007.

Compound ***2*** (Ac-DWSFYLLYYTEF***pT***-NH_2):

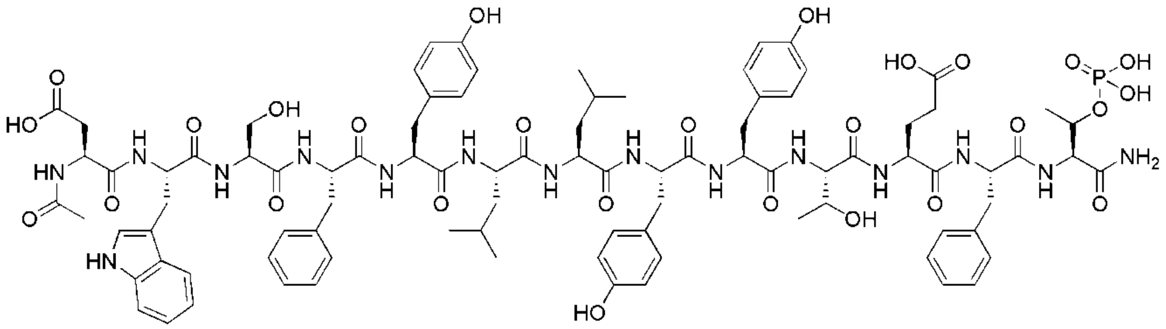

RP-HPLC (Phenomenex Jupiter® 4 μm Proteo 90Å 250 x 4.6 mm): flow rate 1 mL/min; gradient elution (A: H_2O + 0.1% NH_3, B: CH_3CN + 10% A) t=0, A:B 95:5; t=30 75:25; t=32 0:100; Rt 20.6 min (94% purity).

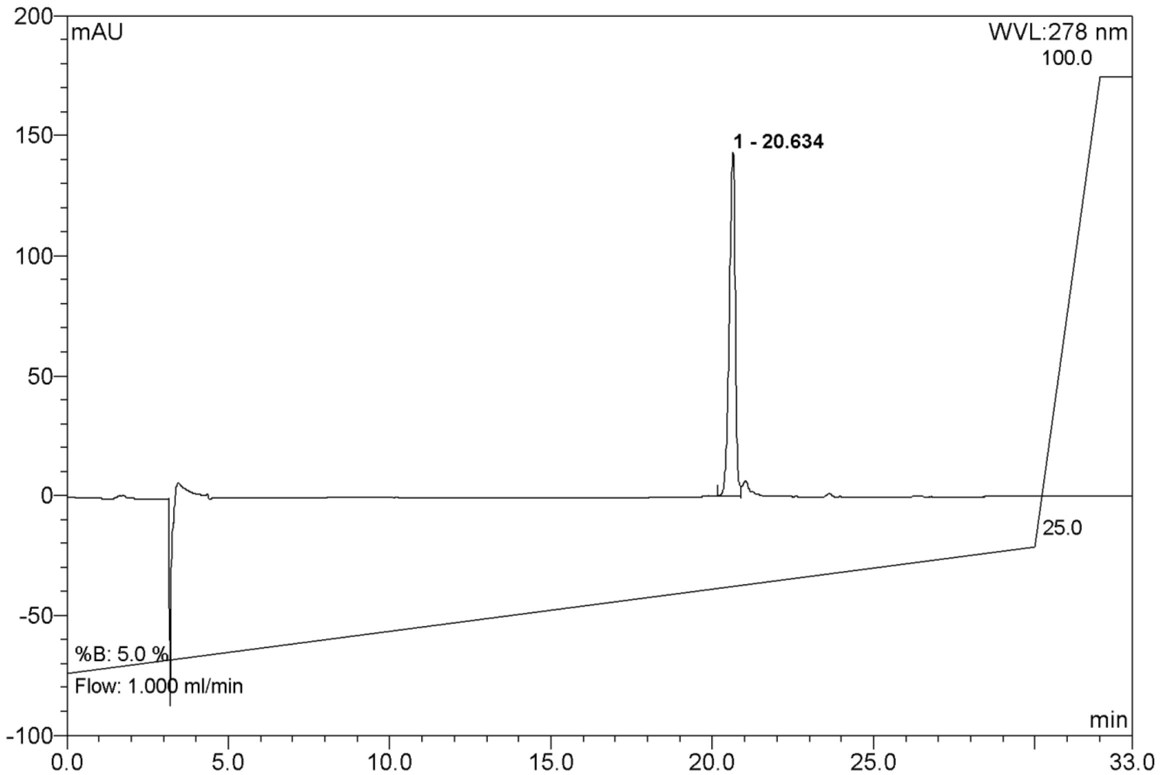

MS (ESI) m/z calcd for $C_{90}H_{112}N_{15}O_{27}P$ $[M-2H]^{2-}$: 932.8800, found 932.8801.

Compound ***3*** (Ac-DWSFYLLYY***pT***EFT-NH$_2$):

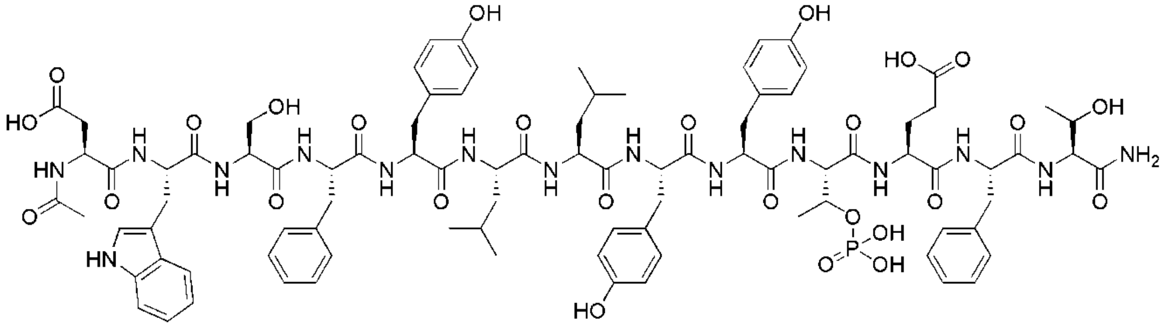

RP-HPLC (Phenomenex Jupiter® 4 µm Proteo 90Å 250 x 4.6 mm): flow rate 1 mL/min; gradient elution t=0, A:B 95:5 ; t=30 75:25 ; t=32 0:100 ; Rt 27.5 min (91% purity).

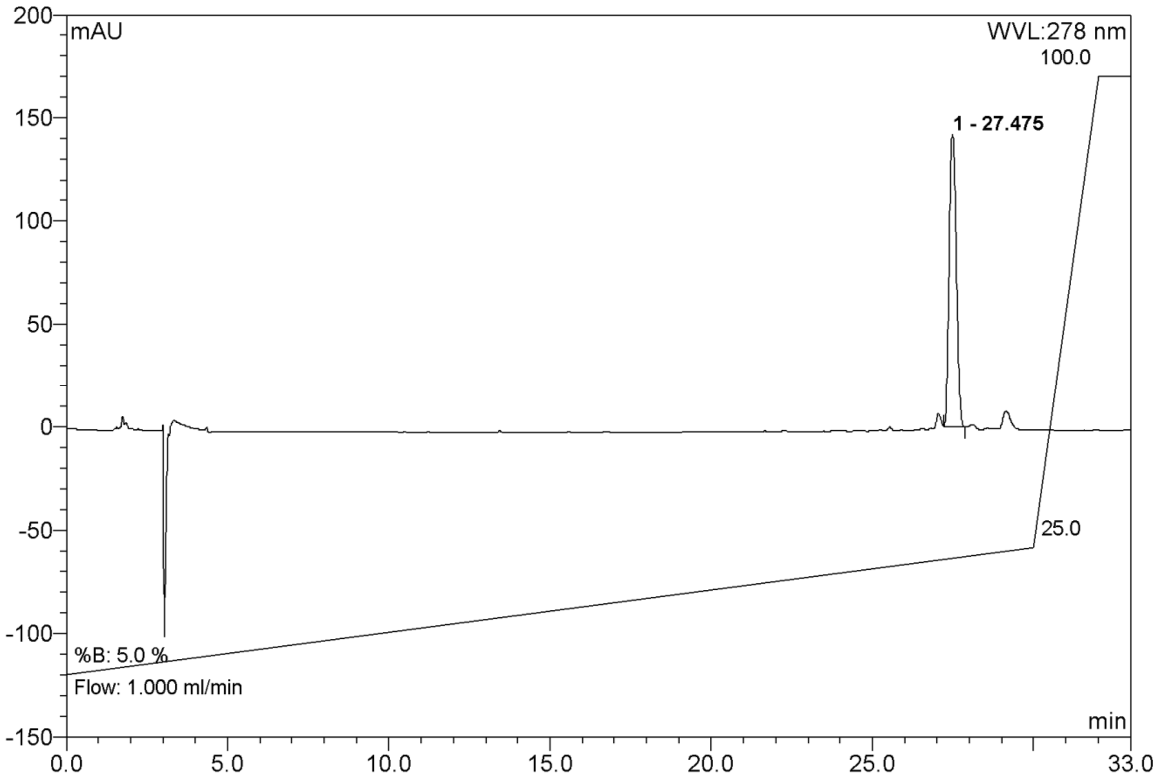

MS (ESI) m/z calcd for $C_{90}H_{112}N_{15}O_{27}P$ $[M-2H]^{2-}$: 932.8800, found 932.8840.

Compound ***4*** (Ac-DWSFYLLY***pY***TEFT-NH_2):

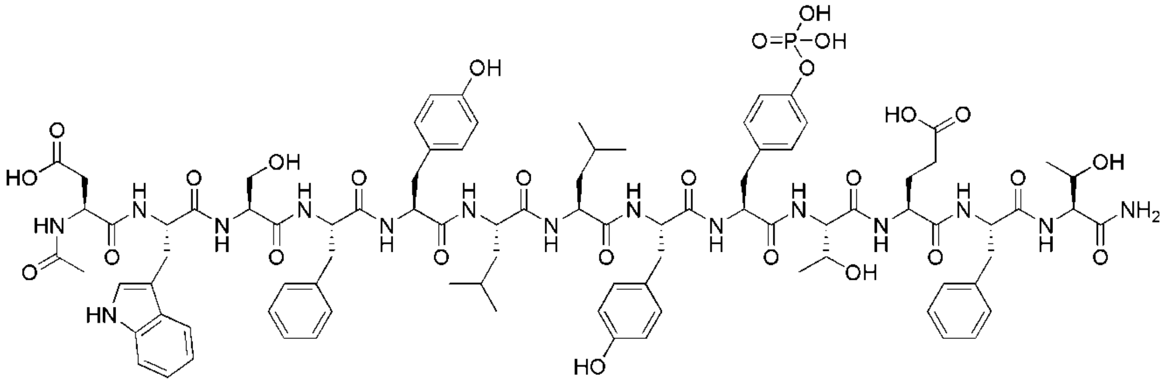

RP-HPLC (Phenomenex Jupiter® 4 µm Proteo 90Å 250 x 4.6 mm): flow rate 1 mL/min; gradient elution t=0, A:B 95:5 ; t=30 75:25 ; t=32 0:100 ; Rt 21.4 min (92.5% purity).

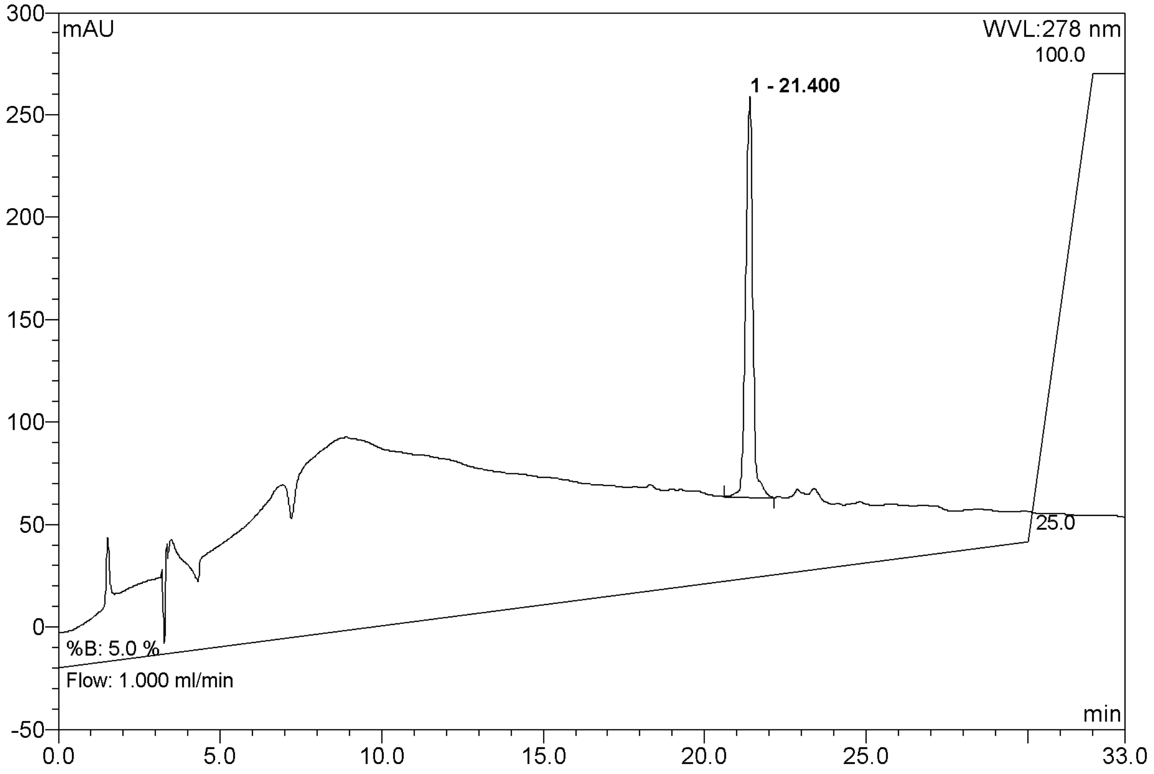

MS (ESI) m/z calcd for $C_{90}H_{112}N_{15}O_{27}P$ $[M-2H]^{2-}$: 932.8800, found: 932.8775.

Compound ***5*** (Ac-DWSFYLL***pY***_Y_YTEFT-NH$_2$):

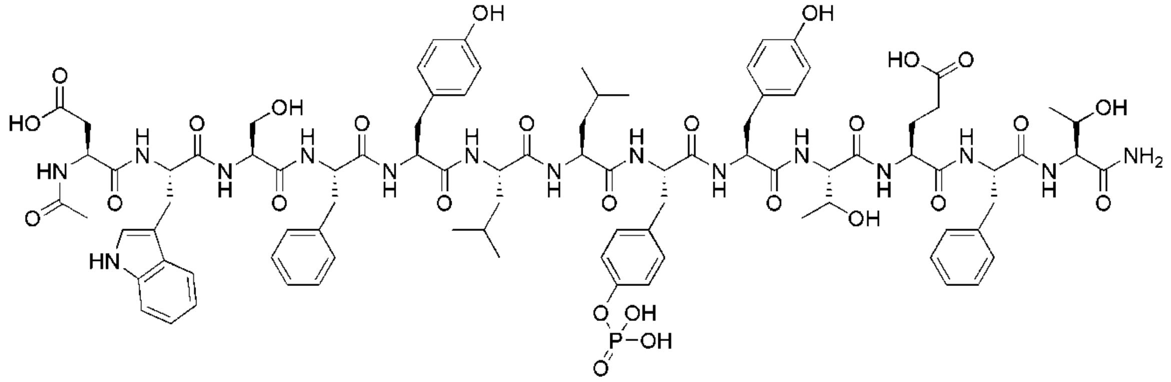

RP-HPLC (Phenomenex Jupiter® 4 µm Proteo 90Å 250 x 4.6 mm): flow rate 1 mL/min; gradient elution t=0, A:B 95:5 ; t=30 75:25 ; t=32 0:100 ; Rt 23.8 min (87.5% purity).

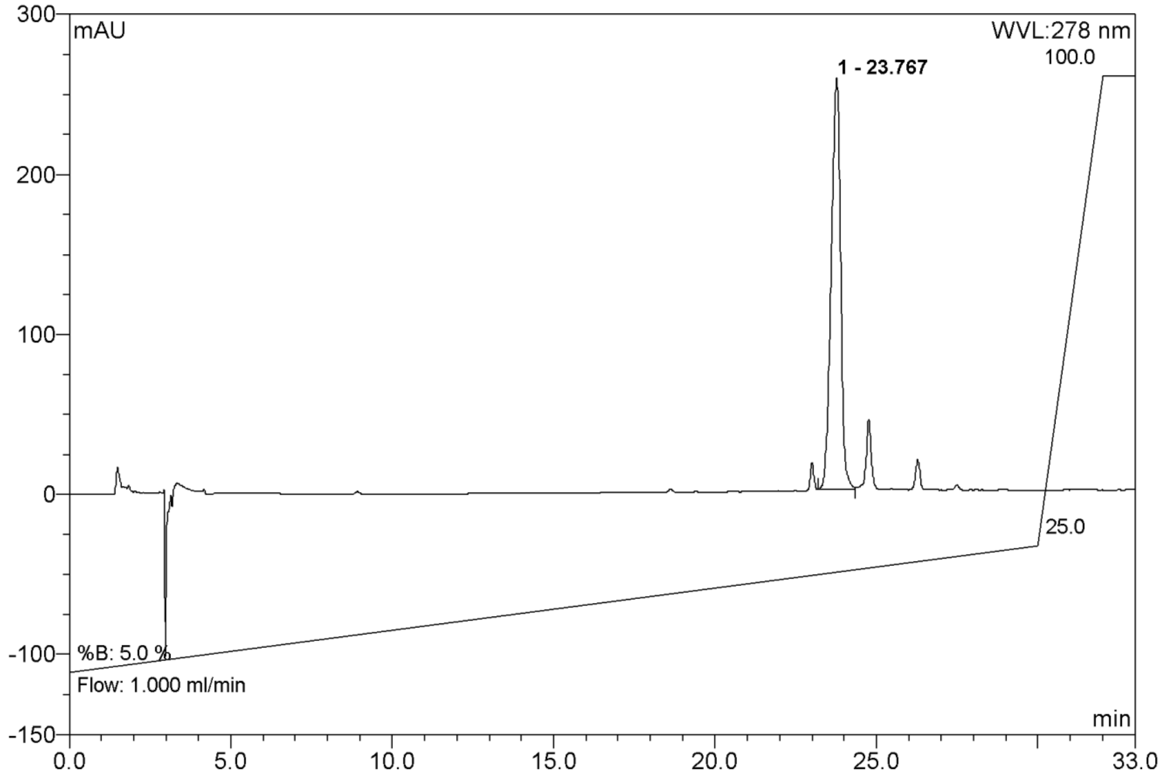

MS (ESI) m/z calcd for $C_{90}H_{112}N_{15}O_{27}P$ $[M-2H]^{2-}$: 932.8800, found 932.8783.

Compound ***6*** (Ac-DWSF***pY***LLYYTEFT-NH_2):

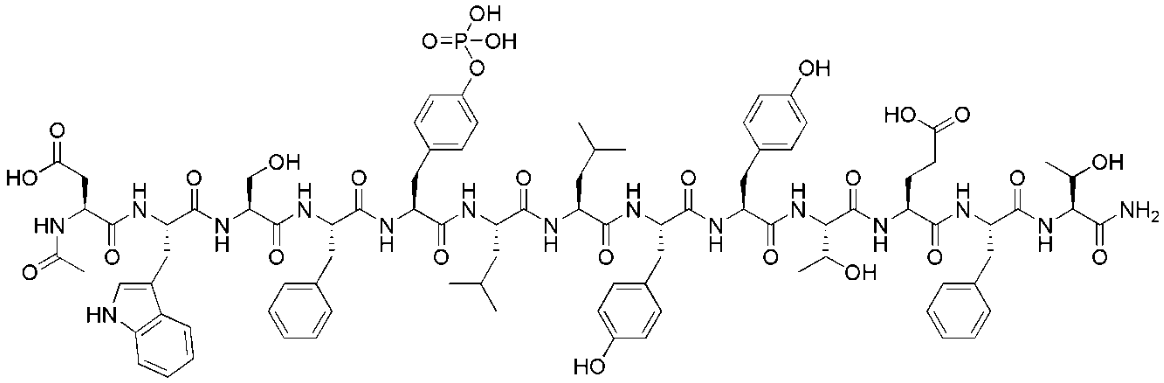

RP-HPLC (Phenomenex Jupiter® 4 µm Proteo 90Å 250 x 4.6 mm): flow rate 1 mL/min; gradient elution t=0, A:B 95:5 ; t=30 75:25 ; t=32 0:100 ; Rt 25.4 min (90% purity).

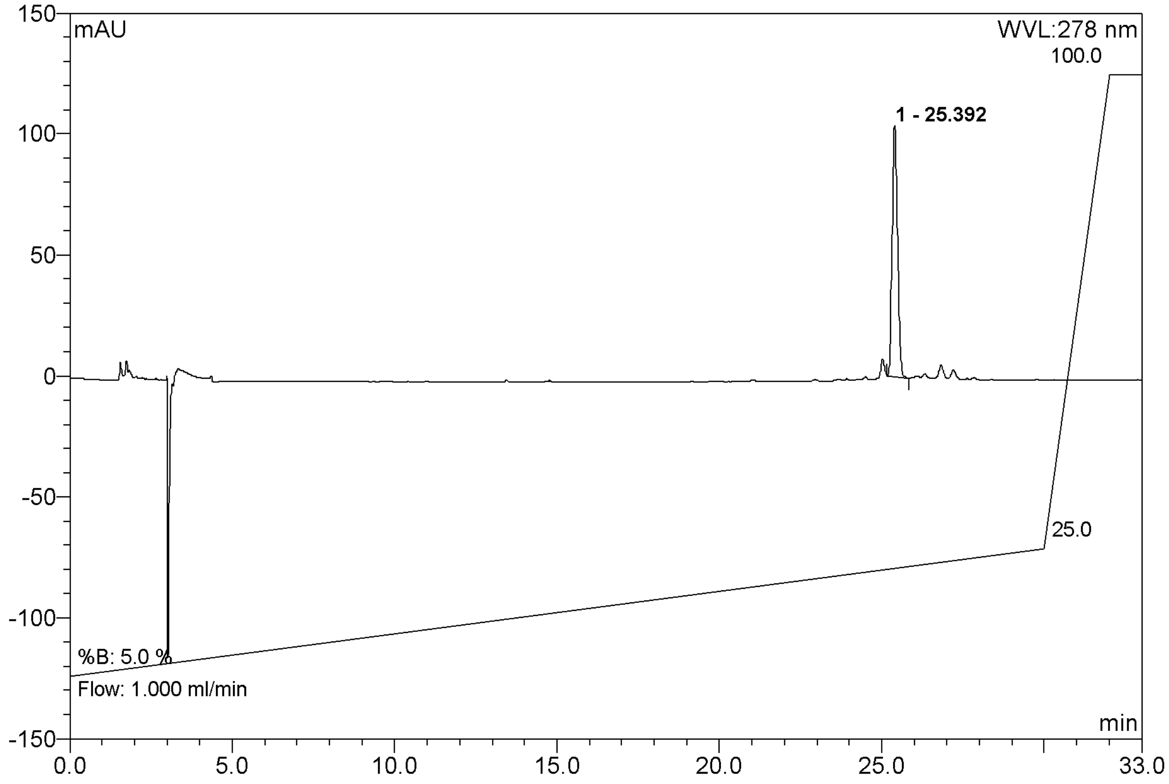

MS (ESI) m/z calcd for $C_{90}H_{112}N_{15}O_{27}P$ $[M-2H]^{2-}$: 932.8800, found 932.8798.

Compound ***7*** (Ac-DW***pS***FYLLYYTEFT-NH_2):

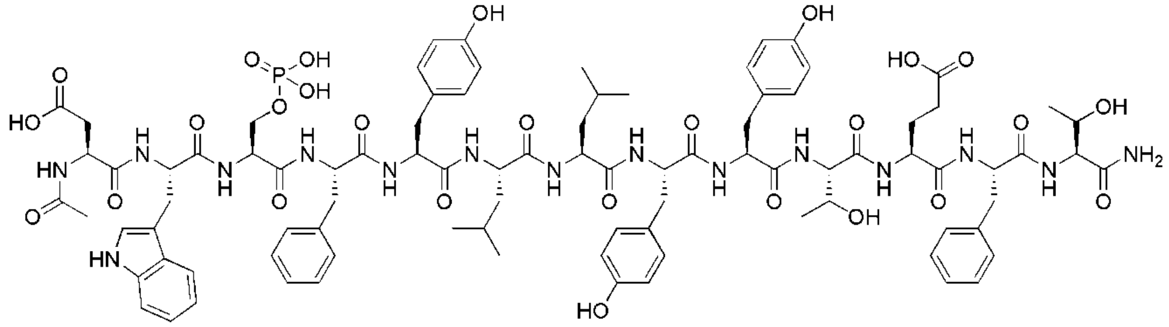

RP-HPLC (Phenomenex Jupiter® 4 µm Proteo 90Å 250 x 4.6 mm): flow rate 1 mL/min; gradient elution t=0, A:B 95:5 ; t=30 75:25 ; t=32 0:100 ; Rt 21.4 min (95% purity).

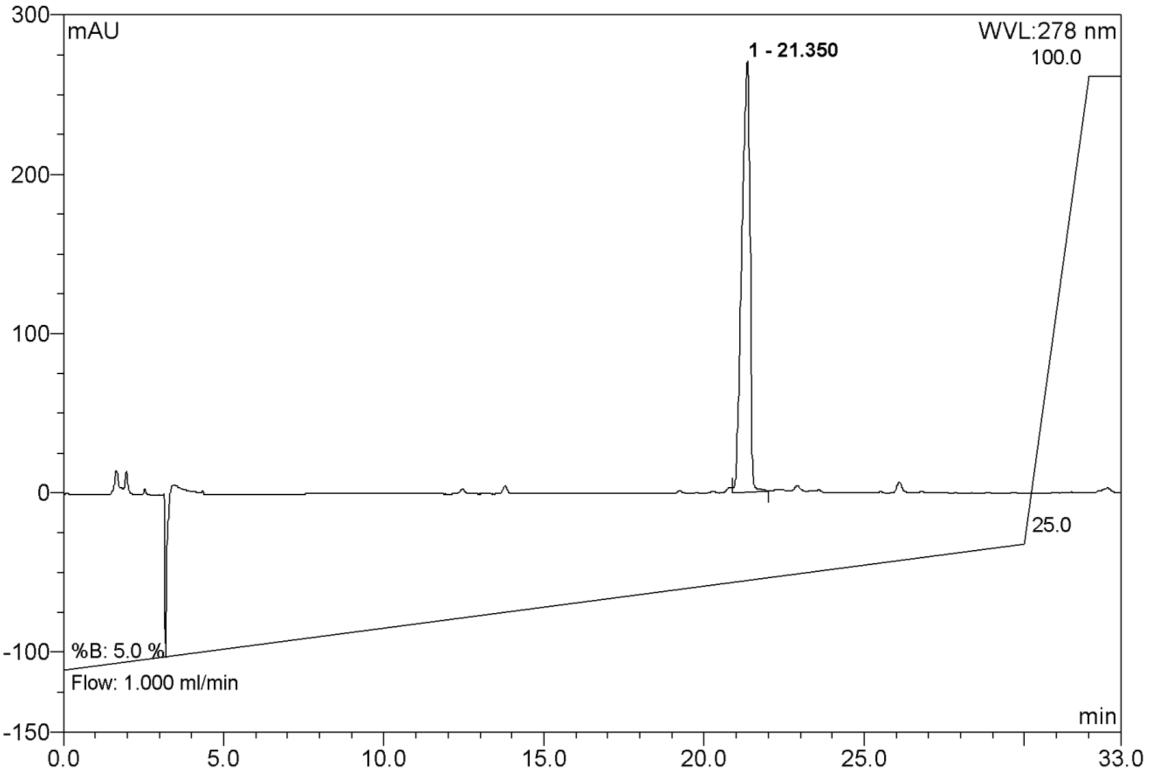

MS (ESI) m/z calcd for $C_{90}H_{112}N_{15}O_{27}P$ $[M-2H]^{2-}$: 932.8800, found 932.8781.

Compound ***8*** (Ac-DW***pS***FYLLYY***pT***EFT-NH_2):

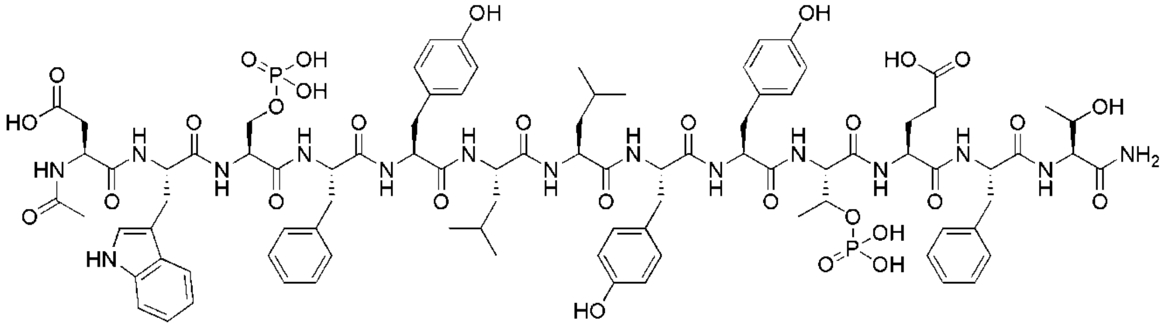

RP-HPLC (Phenomenex Jupiter® 4 µm Proteo 90Å 250 x 4.6 mm): flow rate 1 mL/min; gradient elution t=0, A:B 95:5 ; t=30 75:25 ; t=32 0:100 ; Rt 19.1 min (96% purity).

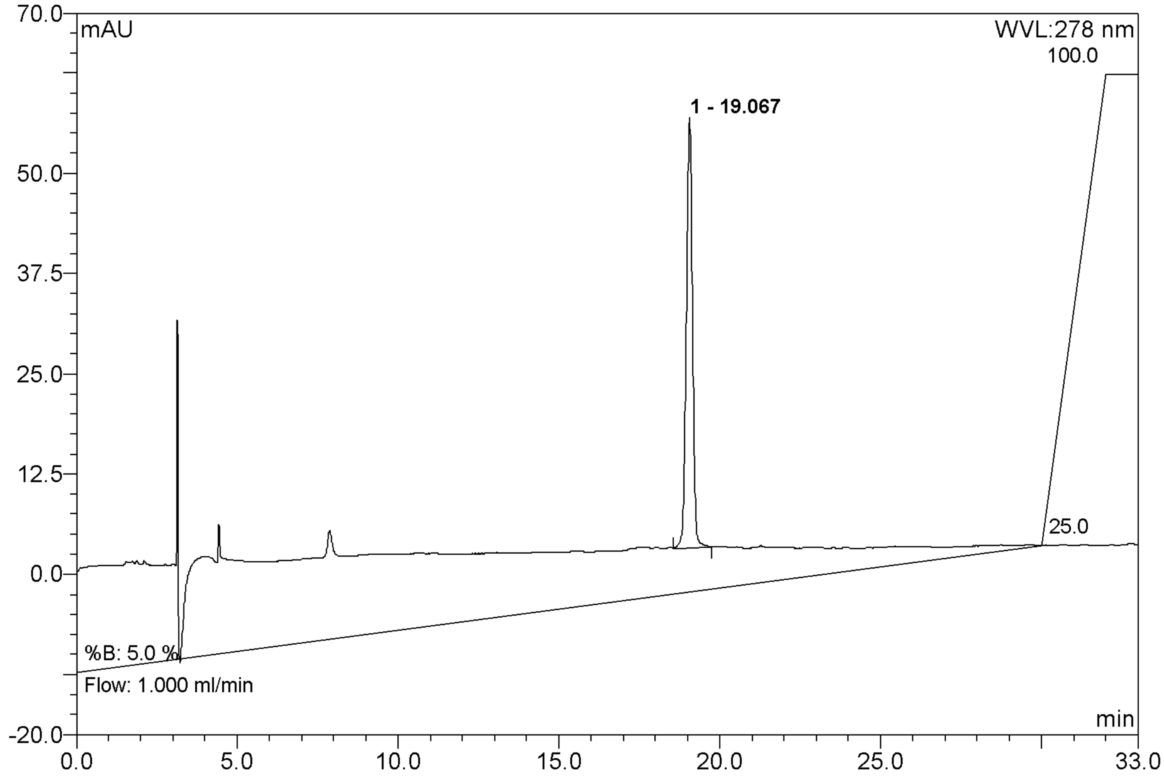

MS (ESI) m/z calcd for $C_{90}H_{113}N_{15}O_{30}P_2$ $[M-2H]^{2-}$: 972.8632, found 972.8645.

2. Determination of Aggregation Properties

Average t_{50} values and standard deviations obtained from the normal distribution.

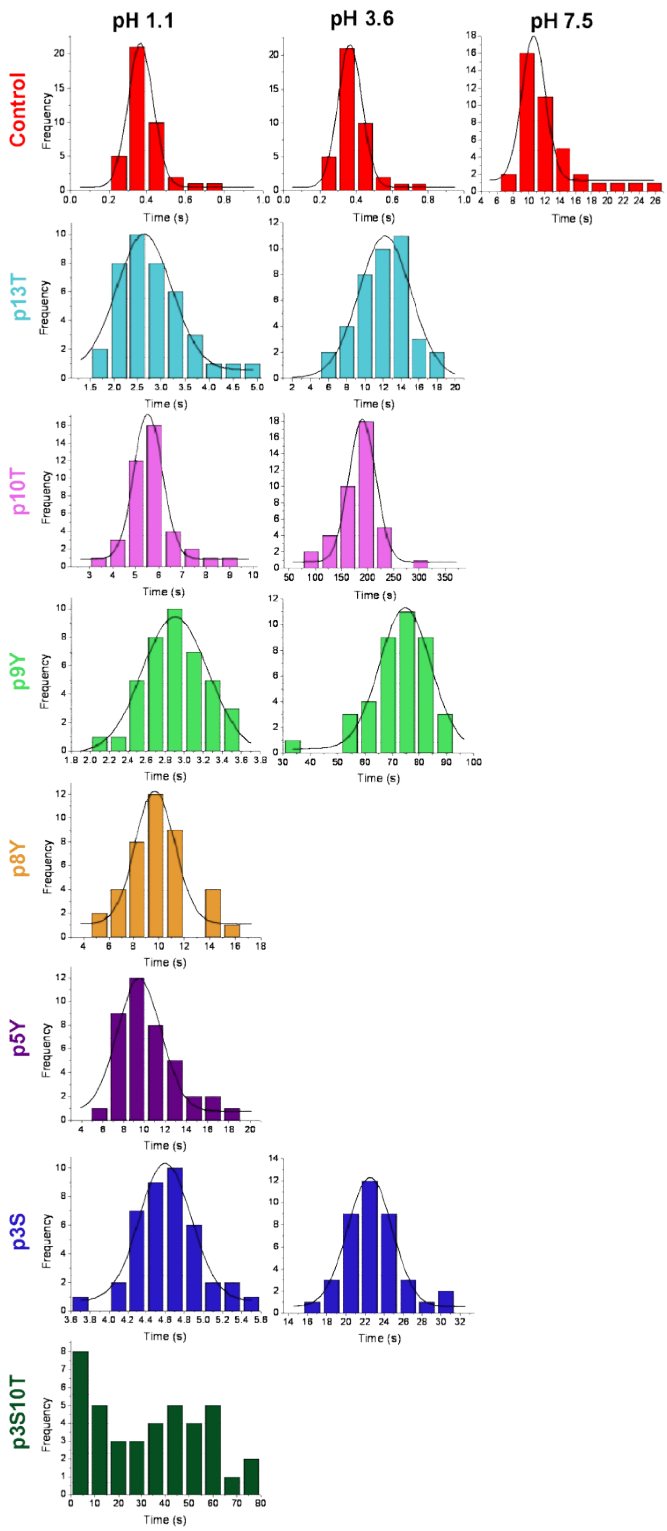

Figure S2. Histogram showing the t_{50} values of each peptide described in Table 1 at pH 1.1, 3.6 and 7.5. The same colour scheme is used as in Table 1. Where no data are shown the peptide did not form fibrils within 4

days. The black solid lines show fits to a Gaussian curve. For control at pH 1.1 and 3.6, the t_{50} was estimated from the amplitude of ThT fluorescence intensity reached in the dead time of the experiment.

Summary of the t_{50} values.

Peptide	pH 1.1		pH 3.6		pH 7.5	
	Mean t_{50} (s)	Standard deviation (s)	Mean t_{50} (s)	Standard deviation (s)	Mean t_{50} (s)	Standard deviation (s)
**Control**	3.7×10^{-1}	3.0×10^{-3}	4.8×10^{-1}	6.0×10^{-3}	10.6	1.8×10^{-1}
**p13T**	2.6	5.7×10^{-2}	12.2	2.8×10^{-1}	$> 10^{5}$	/
**p10T**	5.5	3.2×10^{-2}	190.2	2.4	$> 10^{5}$	/
**p9Y**	2.9	2.3×10^{-2}	74.8	7.7×10^{-1}	$> 10^{5}$	/
**p8Y**	9.7	2.7×10^{-1}	$> 10^{5}$	/	$> 10^{5}$	/
**p5Y**	9.5	2.5×10^{-1}	$> 10^{5}$	/	$> 10^{5}$	/
**p3S**	4.6	2.2×10^{-2}	22.5	1.4×10^{-1}	$> 10^{5}$	/
**p3S10T**	*	*	$> 10^{5}$	/	$> 10^{5}$	/

Table S1. Summary of the averaged t_{50} values for the peptide variants at pH 1.1, 3.6 and 7.5, obtained from the Gaussian distribution shown in Figure S2. Peptides showing no ThT signal and no visible fibrils at the end of the incubation are shown with a t_{50} value of >4 days. * The t_{50} values from the 40 replicates were too scattered to determine an average value.

Electron Microscopy.

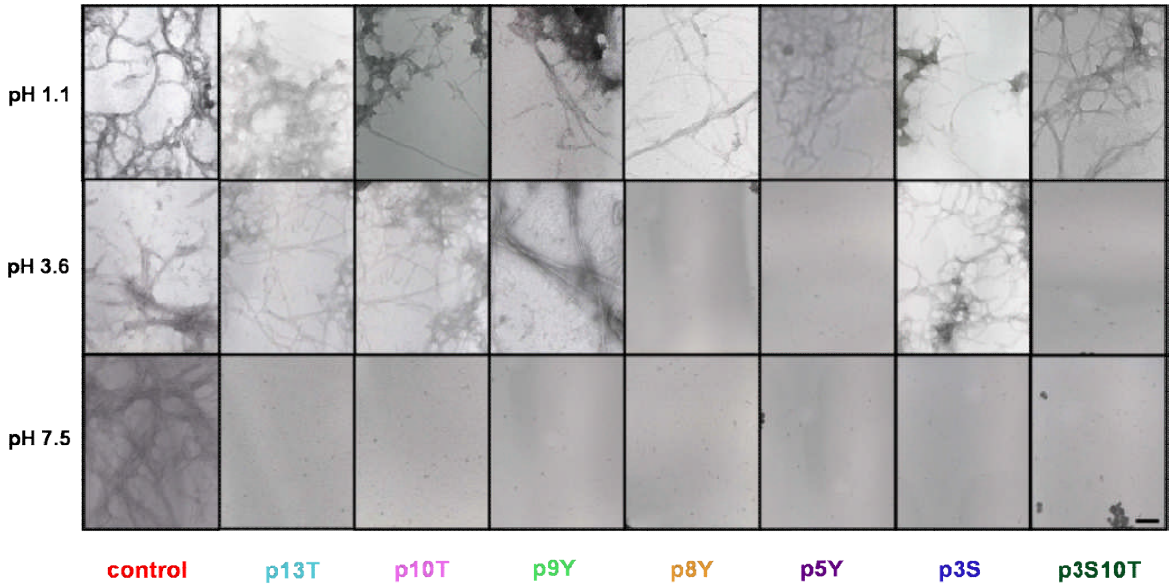

Figure S3. Negatively stained electron micrographs of the end products of fibril formation for the **control** peptide and its phosphopeptide variants at pH 1.1, 3.6 and 7.5, after incubation for 4 days at 30°C, 200 rpm. The scale bar represents 100 nm.

3. MD simulations of p5Y, p8Y and p9Y

Analysis of the MD trajectories

The trajectories generated were viewed with VMD and used to produce the snapshots presented here. The number of water molecules present inside the β-sheet interface over the duration of the unrestrained run was calculated using *ptraj* and a C++ program.

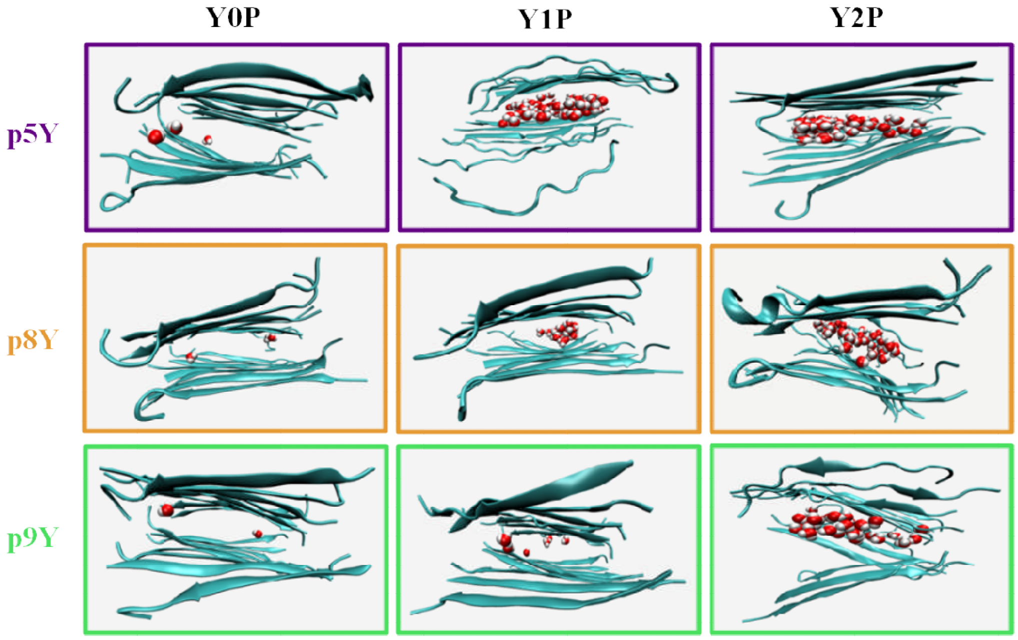

Figure S4. Snapshots highlighting the position of the water molecules found in the inter-sheet interface of the steric zippers **p5Y**, **p8Y** and **p9Y** at the end of the simulations run in explicit solvent. The different protonation states of the phosphate group are shown as Y0P (0), Y1P (-1) and Y2P (-2).
